# High‐Efficiency Quantum Dot Permeable Electrode Light‐Emitting Triodes for Visible Light Communications and on‐Device Data Encryption

**DOI:** 10.1002/adma.202503189

**Published:** 2025-05-30

**Authors:** Seungmin Shin, Hyungdoh Lee, Wonbeom Lee, Seungwoo Lee, Kyung‐geun Lim, Himchan Cho

**Affiliations:** ^1^ Department of Materials Science and Engineering Korea Advanced Institute of Science and Technology (KAIST) Daejeon 34141 Republic of Korea; ^2^ Graduate School of Semiconductor Technology School of Electrical Engineering Korea Advanced Institute of Science and Technology (KAIST) Daejeon 34141 Republic of Korea; ^3^ Korea Research Institute of Standards and Science (KRISS) Daejeon 34113 Republic of Korea

**Keywords:** data encryption, light‐emitting triodes, quantum dot, visible‐light communication

## Abstract

Visible‐light communication (VLC) is a promising technology for alleviating data traffic and spectrum allocation problems. Traditional optoelectronic devices such as light‐emitting diodes (LEDs) are crucial components of VLC systems. However, two‐terminal devices are limited in their functionality and integration capabilities. Thus, a third permeable electrode (PE) is incorporated for high‐efficiency quantum‐dot PE light‐emitting triodes (PeLETs), with a maximum external quantum efficiency of 17.4% and luminance exceeding 29,000 cd m‐2. Then, we elucidate the interplay between the resistor–capacitor circuit and the charge injection process using transient electroluminescence measurements. The expanded functionalities allow the simultaneous modulation of two input data streams within a single device. The PeLETs enhance data throughput and transmission capacity through dual‐channel communication. Furthermore, on‐device data encryption is achieved using the concept of interference in the data transmission process. Single‐device data modulation using PeLETs provides a novel concept for on‐device data encryption for next‐generation, highly secure VLC systems.

## Graphical abstract

A novel three‐terminal PeLET device vertically integrated through a permeable electrode expands additional functionality beyond conventional two‐terminal devices. the specific mechanism is elucidated of E‐field penetration from the integrated capacitor unit to the LED via transient electroluminescence measurements. The PeLET device enables dual‐channel data modulation and on‐device encryption for secure visible light communication.

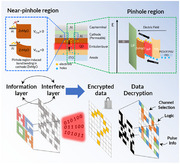

## 1 Introduction

Traditional two‐terminal optoelectronic devices such as light‐emitting diodes (LEDs),^[^ ^1^, ^2^, ^3^ ^]^ solar cells,^[^ ^4^ ^]^ and photodiodes^[^ ^5^, ^6^ ^]^ are essential components of various integrated optoelectronic systems. However, their two‐terminal configurations impose challenges on providing additional functionalities^[^ ^7^ ^]^ and achieving high‐density integrated circuitries.^[^ ^8^, ^9^ ^]^ For example, to provide display panels with a switching functionality, the LED pixels must be integrated with field‐effect transistors and capacitors.^[^ ^10^, ^11^ ^]^ To expand device functionality and diversity, a promising strategy is to transform traditional two‐terminal devices into three‐terminal structures. Recently, three‐terminal devices with a permeable electrode (PE) installed between two vertically stacked device units (e.g., LED/capacitor) have been developed to provide additional functionality to LEDs,^[^ ^8^, ^9^, ^12^ ^]^ solar cells,^[^ ^13^ ^]^ Schottky diodes,^[^ ^14^ ^]^ and neuromorphic devices.^[^ ^15^, ^16^ ^]^ Through its pinholes, the PE allows charge carriers to flow between units, thereby connecting the electrical signals and creating a unified architecture. This approach has demonstrated potential to enhance device performance,^[^ ^17^ ^]^ integration density,^[^ ^8^, ^9^ ^]^ and functionality.^[^ ^18^ ^]^

Among two‐terminal devices, LEDs are a potential contributor to next‐generation wireless optical communication, particularly visible‐light communication (VLC). VLC, also known as light fidelity (Li‐Fi), addresses the growing demand for high‐speed wireless communication^[^ ^19^, ^20^, ^21^ ^]^ by offering a wide bandwidth (400–800 THz), thereby alleviating data traffic and avoiding spectrum allocation competition.^[^ ^21^, ^22^ ^]^ Additionally, VLC is biocompatible, thus extending its applicability to fields such as e‐skin health monitoring^[^ ^23^, ^24^ ^]^ and high‐speed indoor communication.^[^ ^22^, ^24^ ^]^ Recently, III–V inorganic LEDs^[^ ^25^, ^26^, ^27^, ^28^ ^]^ and organic LEDs^[^ ^29^, ^30^, ^31^ ^]^ have been studied as light sources for high‐speed VLC. Key characteristics of LEDs for enhancing data transmission include rapid switching between binary states, and a narrow emission spectrum, which increases the number of available channels for parallel multichannel communications. InP colloidal quantum dots (QDs) exhibit a combination of properties that are well suited for these requirements, including non‐toxicity, narrow full width at half maximum (FWHM), size‐dependent bandgap tunability, and rapid photoluminescence (PL) decay. However, despite these advantages, their application in VLC systems have recieved limited attention thus far.

As VLC adoption expands alongside increasing data traffic, it becomes essential not only to support high‐speed transmission but also to ensure secure data handling, encompassing integrity, confidentiality, and authentication. To this end, various encryption strategies have been proposed, including encrypting the transmitted input signal byintegrating it with random noise signals, such as pseudo‐random binary sequences.^[^ ^32^ ^]^ Other methods include dynamic spectral encryption utilizing photonic crystals,^[^ ^33^ ^]^ meta‐surfaces,^[^ ^34^ ^]^ or chemical spectral variation,^[^ ^35^, ^36^ ^]^ which manipulate optical properties to conceal information. These approaches often require complex decoding schemes, and the encrypted data remain unintelligible without the appropriate decryption key,^[^ ^37^ ^]^ where higher key complexity corresponds to enhanced security.

**Figure 1:**
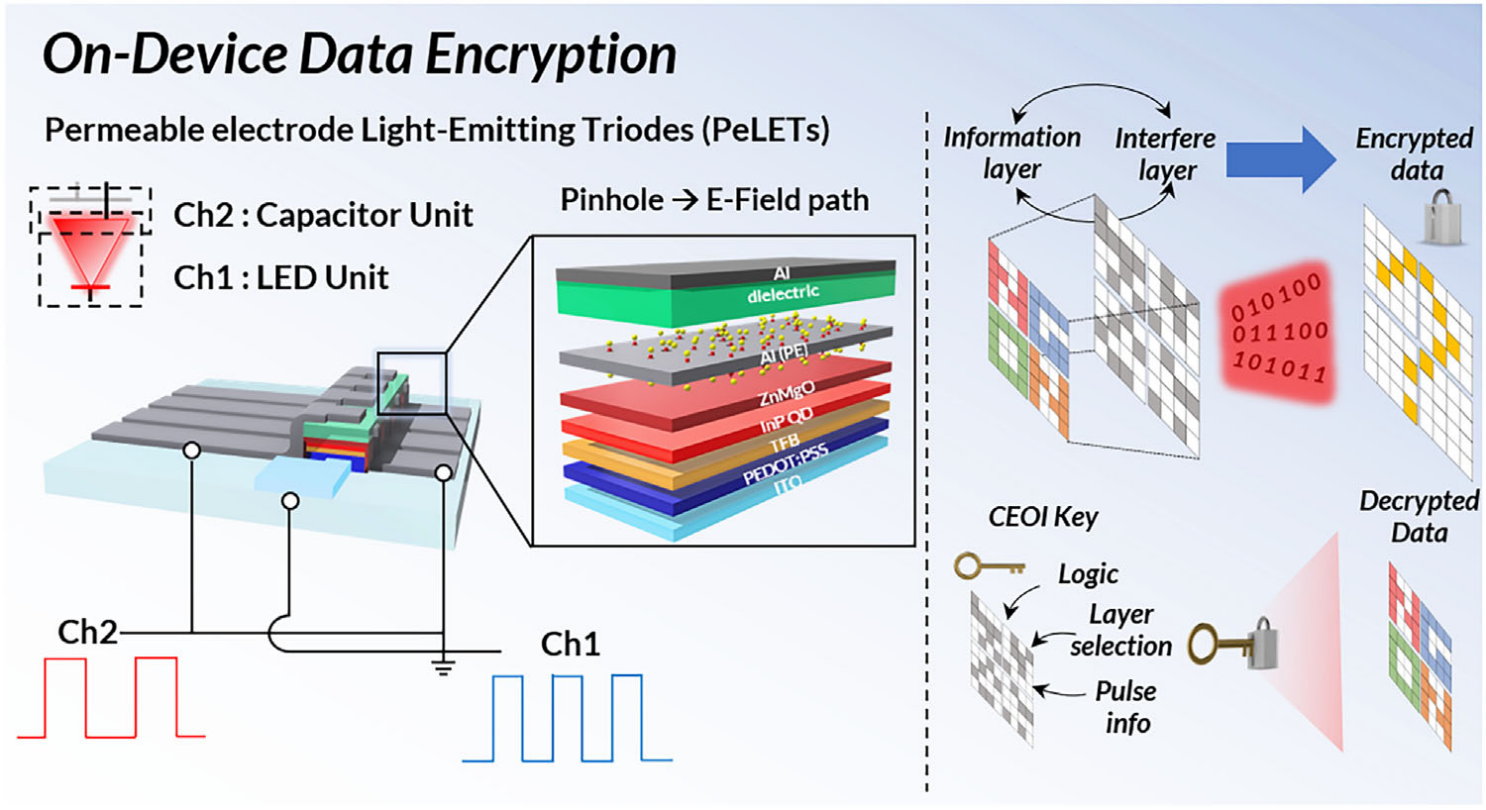
Schematics. PeLET structure and single‐device data modulation through dual‐channel input (left); on‐device data encryption (right).

Herein, we introduce a single‐device data modulation and encryption approach for efficient and secure VLC by leveraging colloidal QDs and a permeable electrode light‐emitting triode (PeLET) architecture. Unlike conventional multi‐component encryption platforms, the PeLET offers a compact and integrable solution by enabling on‐device data processing. Equipped with a PE in addition to the conventional two‐terminal structure (Figure S1, Supporting Information), PeLETs enable simultaneous modulation of two input data streams by acting as optical logic gates for dual‐channel data transmission and on‐device data encryption (**Figure** **1**). This expanded functionality enables secure data encoding through interference layers. The PeLETs incorporate QDs as emission layers (EMLs) and achieve a maximum external quantum efficiency (EQE) of 17.4%, with luminance exceeding 29,000 cd m^−2^. This study demonstrates the potential of PeLET as a novel platform for integrated optical communication and encryption, offering a new paradigm for secure, high‐speed VLC using QD‐based optoelectronic devices.

## 2 Results

### 2.1 PeLET Fabrication and Characterization

The PeLET architecture can be considered a vertically stacked integration of two distinct devices, i.e., a capacitor and an LED, interconnected by a perforated middle electrode, i.e., a PE. With a conventional solid (i.e., non‐perforated) metal electrode, when an electric field (E‐field) is applied, the free electrons within the metal redistribute themselves, effectively blocking the field. Therefore, the E‐field from the capacitor unit cannot penetrate the LED unit.^[^ ^38^ ^]^ To overcome this electromagnetic shielding effect, nanoscale pinholes can be introduced into the middle electrode, thus forming a PE, to facilitate the integration of the two units and, whenever a capacitor voltage is applied, allow the electrical field to penetrate the LED unit (Figure S2, Supporting Information).

Various approaches to constructing permeable middle electrodes have been employed, such as direct pinhole patterning,^[^ ^12^ ^]^ porous Ag nanowire networks,^[^ ^13^, ^14^, ^15^ ^]^ and ultrathin metal films^[^ ^8^, ^17^, ^18^, ^39^ ^]^ (<15 nm). However, directly patterning the middle electrode can cause significant damage to the underlying layers, whereas Ag nanowire electrodes present challenges to efficiency owing to their high work function (≈4.46 eV)^[^ ^14^ ^]^ and low reflectance (≈3%).^[^ ^13^ ^]^ Meanwhile, excessively thin metal electrodes can suffer from a very high resistance, also making them unsuitable (Figure S3a, Supporting Information). To achieve high‐efficiency PeLETs, the PE must have sufficiently low resistance and high reflectance, while avoiding damage to the underlying layers during deposition. Additionally, a balanced injection of electrons and holes is essential for efficient electroluminescence within the LED unit. In our PeLET architecture (Figure S1, Supporting Information), the PE serves as a cathode; therefore, engineering both the electron transport layer (ETL) and the sheet resistance of the PE is critical, as further detailed in the performance enhancements shown in Figure S3 and S4 (Supporting Information).

Aluminum growth typically follows Volmer–Weber growth, where islands form on the substrate owing to the interplay between adatoms and substrate–adatom interactions.^[^ ^40^ ^]^ As the growth progresses, these islands tend to connect, reducing the number of voids and decreasing the resistivity of the film with increasing thickness.^[^ ^39^, ^41^ ^]^ As shown in Figure S3 (Supporting Information), a 25‐nm aluminum layer exhibits a relatively low sheet resistance (1.3 Ω sq^−1^), which enables efficient electron injection into the EML. As demonstrated in Figure S3 (Supporting Information), this optimized PE thickness (25 nm) preserves the permeability required for electric field penetration from the capacitor unit, while simultaneously ensures both high electroluminescence efficiency and a rapic electric field‐driven response required for VLC applications.

To overcome this, we fabricated the PE via deposition onto a perforated Zn_87.5_Mg_12.5_O (ZnMgO) ETL, which naturally produced pinholes in the PE. Prior to that, the pinholes in the ZnMgO layer were induced by a dimethyl sulfoxide (DMSO) additive in the ZnMgO nanoparticle dispersion. This effect arises from surface tension differences caused by Bénard–Marangoni convection, driven by an imbalance in vapor pressure and viscosity between DMSO and ethanol in the co‐solvent system during vaporization (see Supplementary Note 1 for details). The pinholes in the aluminum middle electrode, which closely matched the perforated morphology of the ZnMgO layer (Figure S2, Supporting Information), resulted in a relatively thick, low‐resistance PE suitable for PeLET applications. The DMSO treatment did not cause significant variations in the electronic and optical characteristics, including the valence band maximum and optical band gap of the ZnMgO ETL (Figure S5, Supporting Information). These properties were preserved because the DMSO treatment primarily influences the pinhole formation and colloidal stability (Figure S6, Supporting Information) of the ZnMgO nanoparticle dispersion and leaves no residues after spin‐coating and subsequent annealing, as confirmed by the absence of S2p peaks in the X‐ray photoelectron spectroscopy (XPS) spectrum (Figure S5, Supporting Information).

**Figure 2:**
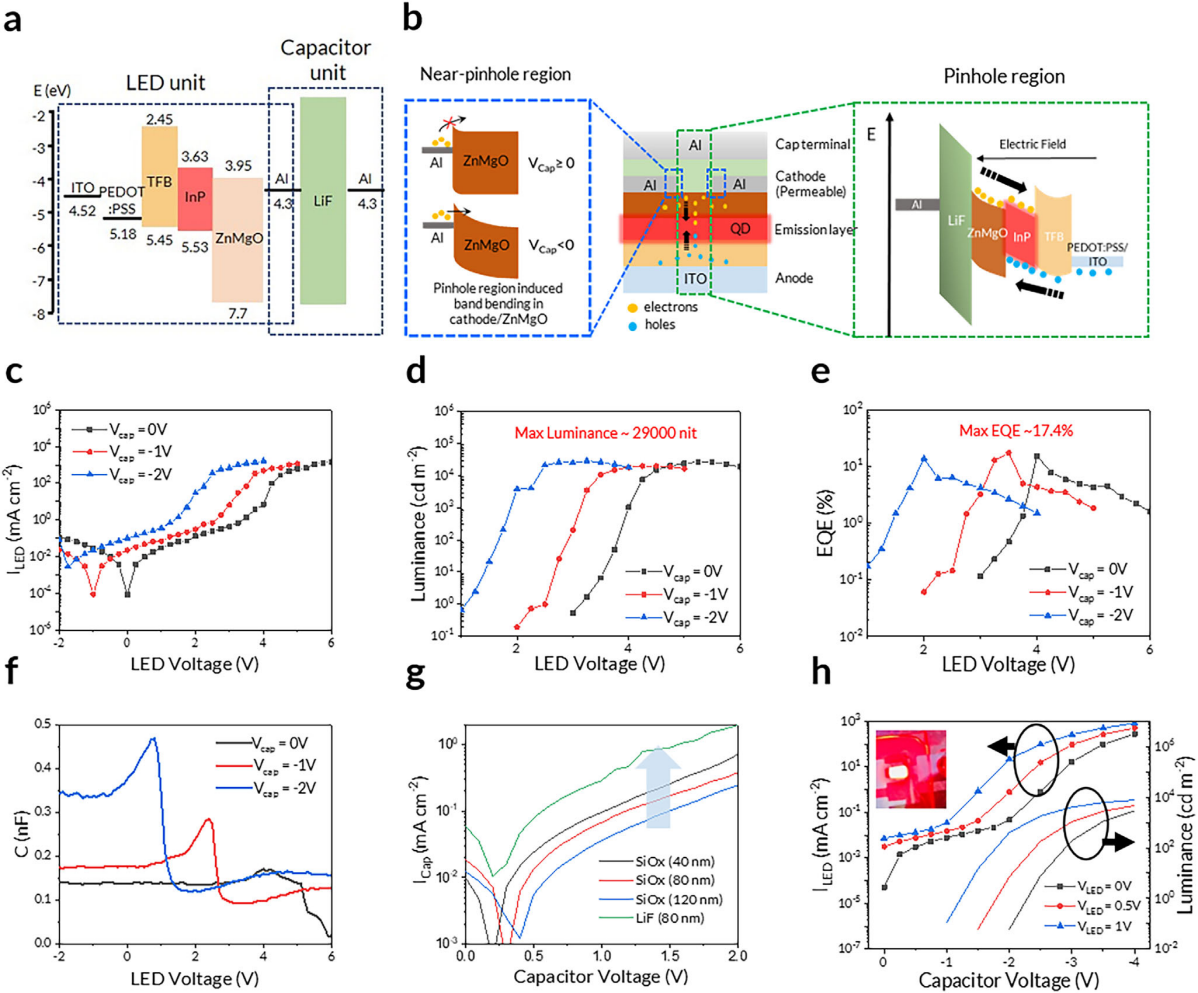
Electrical Characteristics of PeLETs. a) Band diagram. b), Schematic working mechanism (E‐Field penetration) when negative voltage was applied at capacitor unit. Note that the core/shell and ligand structure of emission layer was simplified as a single band. c,d,e), Current density, luminance, and EQE vs. LED voltage (applied bias at LED unit), respectively, under zero and negative capacitor unit bias (0 V, −1 V, −2 V). f), Capacitance vs. voltage at 1 kHz. g), Current density vs. voltage, with capacitance‐dominated region varied by different dielectric and thickness h), Controllability of LED luminance via capacitor unit bias.

We fabricated PeLETs by integrating InP/ZnSe/ZnS QD‐based LED unit and capacitor unit and measured their current–voltage–luminance (I–V–L) characteristics (**Figure** **2**). The choice of QD EML provides several advantages, including a high photoluminescence (PL) quantum yield, narrow emission spectrum, and relatively short PL lifetime.^[^ ^19^, ^42^, ^43^ ^]^ The short PL lifetime enables high‐frequency switching, potentially enhancing the data transmission speed, whereas the narrow spectral linewidth increases the number of available transmission channels for parallel multichannel communication. The band diagram of the PeLET, illustrating the LED–capacitor vertical integration, is shown in Figure 2a. Also, Figure 2b illustrates the proposed operating mechanisms in the pinhole and near‐pinhole regions. In the pinhole region, under a negative *V_Cap_*, the localized E‐field penetrates through the pinholes of the PE, facilitating the injection of both electrons and holes in the LED unit. Specifically, the E‐field induces band bending in the ZnMgO ETL near its interface with the PE, enhancing the electron injection from the PE to the LED unit. The localized E‐field contributes to the enhancement of electron tunneling current in the near‐pinhole region. The detailed device operation mechanism will be further discussed in the transient electroluminescence (TrEL) response section.

The PeLET achieved a maximum EQE of 17.4% and maximum luminance of ≈29,000 cd m^−2^. Its I–V–L characteristics gradually shifted leftward (toward negative *V_LED_*) as the capacitor bias *V_Cap_* was adjusted from 0 V to −2 V (Figure 2c). The direction of the applied bias at LED unit and Cap unit is illustrated in Figure S1 (Supporting Information). It is worth noting that the maximum luminance did not decrease with varying capacitor bias despite the overall shift in the curve (Figure 2d,e). This implies that the electrical equilibrium state of the LED unit was altered by the E‐field generated by the capacitor.

To investigate the relationship between the E‐field from the capacitor and charge injection into the LED unit, the capacitance of the LED unit (*C_LED_*) was measured. C–V measurements show a significant increase in the capacitance, followed by a rapid release, indicating charge accumulation before the flat‐band condition is reached in the LED.^[^ ^44^ ^]^ To clarify the charge accumulation and injection mechanism, a thin LiF charge accumulation layer (1.5 nm) was intentionally added between the QD EML and ZnMgO ETL (Figure S7, Supporting Information). When the forward bias exceeds the turn‐on voltage and the flat‐band condition is achieved, the accumulated charges are injected simultaneously, resulting in a rapid decrease in the capacitance. As shown in Figure 2f, the capacitance overshoot before the flat‐band condition was gradually shifted by the applied *V_Cap_*, indicating that the charge carrier injection in the LED unit was facilitated by the capacitor‐induced E‐field (Figure 2b). In addition, the capacitance at *V_LED_* = 0 V also increases as *V*_Cap_ increase. The capacitance in the LED is governed by two factors: the depletion region capacitance and the capacitance arising from charge distribution.^[^ ^13^, ^44^ ^]^ The initial increase of the capacitance in the C–V curve (measured at *V_LED_* = 0 V) indicates that the penetrating E‐field by *V_Cap_* modulates the charge distribution in the LED unit.

The penetrating E‐field follows the parallel plate capacitor equation (C=εA/d), where ε is the dielectric constant, *A* is the capacitor area, and *d* is the dielectric thickness. Figure S8 (Supporting Information) shows the capacitor current (*I_Cap_*) measured for various dielectric constants and thicknesses, with the region where the *I_Cap_* dominates over the *I_LED_*, as highlighted in Figure 2g. As the capacitance increased with decreasing thicknesses and increasing dielectric constants, the *I_Cap_* also increased. The relationship between *I_Cap_* and *I_LED_* in the PeLEDs is further detailed in Supplementary Note 2. When the capacitor voltage was applied in the absence of pinholes, the *I_LED_* did not change (Note S2 and Figure S21a, Supporting Information), owing to the electromagnetic shielding effect.^[^ ^38^ ^]^ By contrast, the PE enables the integration of a new terminal that provides additional controllability over the characteristics of the LED. For example, control over the LED luminance via capacitor modulation is demonstrated in Figure 2h, and Figure S9 (Supporting Information). The additional terminal, i.e., the capacitor unit, effectively modulates the charge injection and emission characteristics of the LED unit. We developed an optimized PeLET structure that achieve both high EQE and robust modulation by capacitor unit, by combining perforated ZnMgO ETL engineering and PE engineering. This structure allows the PE to function as effectively as a cathode by maintaining sufficient low sheet resistance, while preserving the performance of the LED unit under capacitor‐induced modulation.

### 2.2 Transient Electroluminescence Measurements of PeLETs

**Figure 3:**
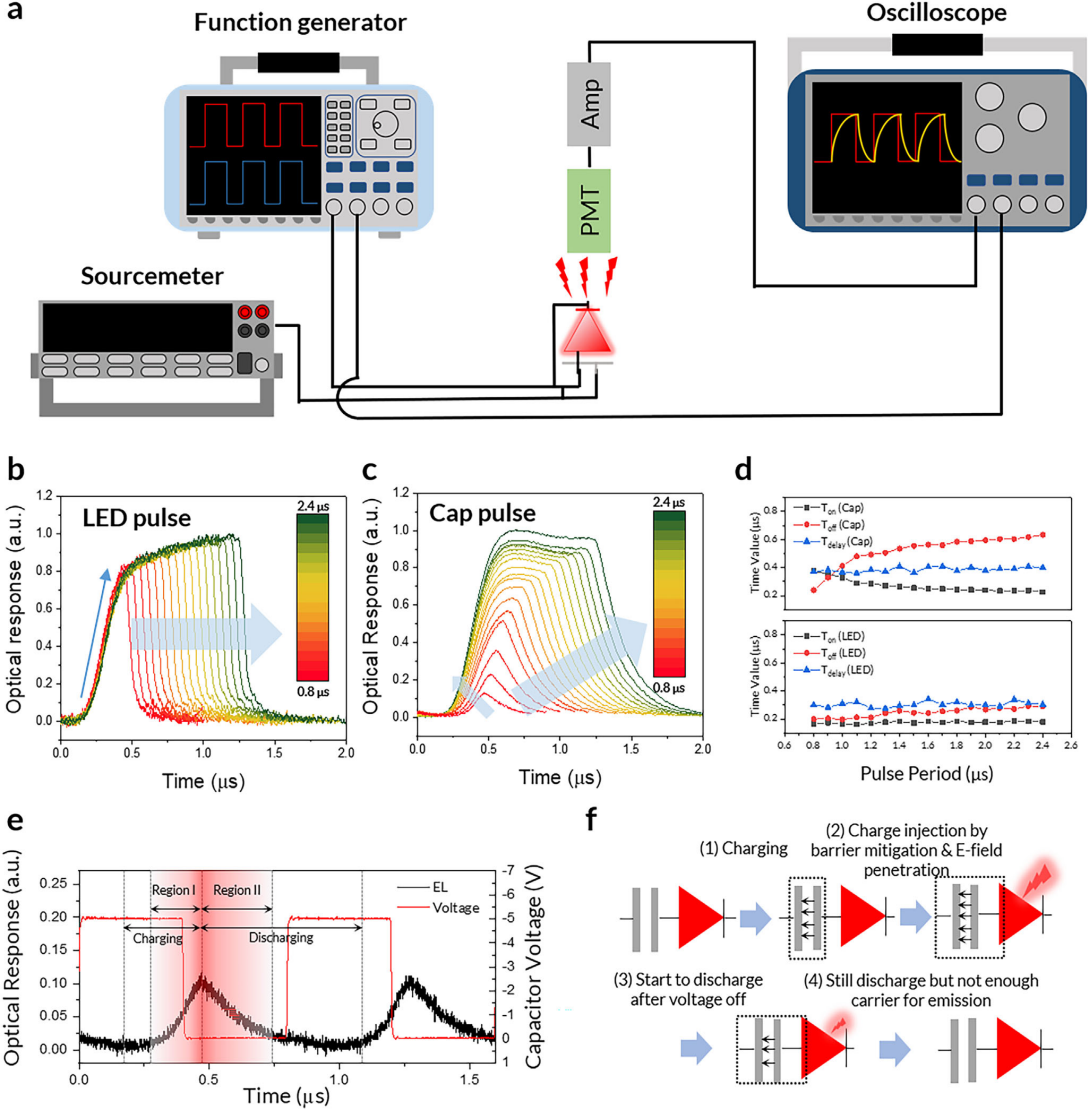
Transient Electroluminescence in PeLETs a), Block diagram for TrEL testing setup specialized to measure three‐terminal PeLET devices. During the TrEL response through electrical pulses at the LED and capacitor units, the DC bias at the opposite side was 0 V. The dielectric layer was 80 nm of SiOx. b,c), TrEL response to pulses ranging from 0.8 to 2.4 µs applied to LED unit (5.0 V) and capacitor unit (‐5.0 V). d), *T_on_*, *T_off_*, and *T_delay_* value tendencies as pulse period increases. *T_on_* and *T_off_* were obtained based on the time value at the threshold level (EL intensity = 0.05) during the on and off process. *T_delay_* was calculated as the maximum point of the 1^st^ derivative (see details in S3, Supporting Information). e), TrEL waveform under Cap pulse operation (period: 0.8 µs, *V_Cap_* = −5 V), showing the influence of charging and discharging effect, resulting in delayed EL response. Region I and II indicate the direct relations between carrier injections and recombination: Region I (Injection > Recombination), Region II (Recombination > Injection) f), Schematic of charging and discharging processes in PeLETs under Cap pulse operation.

To investigate the dynamic behavior of the PeLETs, we characterized their TrEL responses under pulsed operation, as depicted in **Figure** **3**a. A source meter, function generator, photomultiplier tube (PMT), and oscilloscope were used to ensure precise control of the pulses and real‐time monitoring of the EL signals. The PeLET architecture forms an intrinsic resistor–capacitor (RC) circuit, owing to its vertically stacked LED and capacitor units. Its three‐terminal configuration allows for two distinct modes of pulsed operation, depending on the point of electrical contact, i.e., anode‐PE for “LED pulse” and cathode‐PE for “capacitor pulse (Cap pulse)” (Figure 3b,c), enabling the investigation of its charge‐carrier dynamics, particularly the influence of the capacitor unit on EL modulation in the LED unit. In a conventional LED, EML carrier dynamics are driven by drift, which arises from the E‐field, and by diffusion, which is induced by concentration differences. In TrEL, characteristic times can be categorized into two regions:^[^ ^45^, ^46^ ^]^ the delay time region, which is predominantly affected by drift, and the rising time region, which is influenced by both drift and diffusion. To analyze the characteristics induced by Cap pulse operation in PeLETs, we define two additional characteristic times: *T_on_* and *T_off_*. Here, *T_on_* is the time at which the normalized EL intensity exceeds a threshold level, and *T_off_* is the time at which it drops below the threshold, corresponding to the literal turning on and off of the EL response (see Supplementary Note 3 for calculation details). Figure 3b,c illustrate the TrEL intensity waveforms under LED and Cap pulses, respectively, with varying pulse periods ranging from 0.8 to 2.4 µs (50% duty cycle). When driven by the LED pulse, the PeLET shows short and nearly constant *T_on_* (≈0.18 µs) and *T_delay_* (≈0.30 µs), indicating direct carrier injection into the LED unit (Figure 3d). By contrast, for the Cap pulse, *T_on_* (0.22–0.38 µs) and *T_delay_* (≈0.39 µs) values are much longer than those at LED pulse. In particular, the *T_off_* value significantly increases from 0.24 to 0.63 µs with increasing the pulse period, primarily due to the greater number of injected carriers, which are responsible for the delayed EL after pulse‐off. However, the increase in *T_off_* becomes less significant at higher pulse periods, suggesting that carrier concentration in the PeLET reaches a saturation level. This saturation effect limits further increases in *T_off_* with longer pulse periods.

These distinct TrEL behavior under Cap pulse operation can be better understood by considering the intrinsic RC circuit characteristics of the PeLET architecture. With the Cap pulse, additional factors come into play in the operation mechanism of the PeLET due to the charging and discharging dynamics of the capacitor unit. Unlike conventional LEDs, the capacitor unit of the PeLET introduces a delayed EL response due to the RC time constant τ (*V*(*t*)  =  *V*_0_ *e* ^−*t*/τ^), as shown in Figure 3e. Specifically, the reduction in EL in region II caused by the recombination of residual carriers during, diffusion is much slower in *T_off_* (Cap) than in *T_off_* (LED), resulting in an extended EL delay. This delay arises from the gradual discharge of the capacitor unit, during which the residual electric field continues to inject carriers into the EML, thereby postponing the complete turn‐off of the LED unit. This behavior highlights a characteristic of PeLETs in which the capacitor unit modulates charge injection and emission in the LED unit through E‐field penetration at the PE. In addition, *T_on_* (Cap) is generally longer than *T_on_* (LED), and gradually decreases with increasing pulse period (Figure 3d). This indicates that the internal E‐field formation is influenced by residual charges remaining from incomplete discharge during short‐pulse operation. These residual charges can partially screen the applied field, slowing down the onset of effective carrier injection. In contrast, longer pulse periods allow sufficient discharge in the preceding cycle, enabling faster field buildup and resulting in a shorter *T_on_*. Additionally, *T_delay_* values, which reflects the drift dynamics in EML show consistency both in the Cap and LED pulse operation, even though Cap pulse modulation is affected by charging in capacitor. These phenomena, relatively high *T_on_* (Cap) and consistent *T_delay_* (Cap) imply that the carrier in the EML follows the charging and drift mechanism, as shown in Figure 3f. Furthermore, the slow relaxation of the EL is also induced by the delayed discharging of the capacitor unit, indicating that the mitigated injection barrier increases slowly to the initial level during the discharging process. Thus, we have inferred the operating mechanism of PeLETs under both DC and pulse‐bias conditions. The E‐field penetrating the PE reduces the injection barrier in the LED unit, and the speed of the mitigation process adheres to the RC circuit nature. Figure S10 (Supporting Information) shows the pulse‐period‐dependent EL intensities measured at various DC bias levels applied to the LED unit. The normalized EL intensities, which accounted for variations across different DC bias levels, were closely aligned (Figure S10f, Supporting Information), suggesting that the RC characteristics affecting the rate of EL increase was influenced by the intrinsic properties of the capacitor units, rather than by LED units.

Additionally, EL overshoots were observed at the corners of the rising edge. Interestingly, a stronger EL overshoot was observed as *V_LED_* increased (Figure S11a, Supporting Information). The variation in the EL overshoot has previously been ascribed to different charge injection and accumulation behaviors, depending on the electronic properties of the charge transport layer and EML.^[^ ^45^, ^46^, ^47^, ^48^, ^49^ ^]^ Similarly, in our PeLET device, the appearance of such overshoots can be attributed to the charging effect, which originates from the unbalanced electron and hole injection dynamics while the E‐field penetrates through the pinholes. During the charging process in the Cap unit, the drift of electrons induced by the E‐field is more dominant than that of holes in the LED unit (Figure S11b, Supporting Information). Thus, due to the sequential injection of electrons and holes, the EML is locally charged and exhibits an overshoot in TrEL. Particularly, when the LED pulse is applied, no overshoot is observed, whereas an overshoot appears under the Cap pulse. This indicates that charge injection driven by the LED unit bias differs fundamentally from that under the Cap unit bias. With the LED unit bias, both electrons and holes are simultaneously influenced by the internal electric field. In contrast, the Cap unit bias exhibits charging and discharging behavior characteristic of the intrinsic RC circuit, along with sequential charge injection facilitated by E‐field penetration. Therefore, the operation mechanism in the Cap pulse modulation is closely correlated with the capacitance of the capacitor unit. Figure S12 (Supporting Information) shows, based on trends in the increment in EL under varying voltages and dielectric thicknesses, that reduced capacitances resulting from thicker dielectrics can help enhance high‐frequency operations by modulating the RC circuit. However, the reduced EL intensities at lower capacitances negatively affect the accuracy of data transmission. While lower capacitance improves modulation speed, this comes at the cost of diminished signal strength, indicating a trade‐off between speed and reliability. Therefore, dielectric engineering and device optimization are therefore crucial for achieving a much higher −3 dB on‐off keying(OOK) bandwidth for high‐speed VLC. The OOK bandwidth characterizes the intrinsic temporal response of the device, by identifying the cutoff frequency where the optical power drops by 3 dB under binary switching (Figure S13, Supporting Information). This measurement reflects the carrier dynamics and temporal response capability of the PeLET, but does not represent the upper limit of the data rate achievable in our system. (See Supplementary Note 4 for further details).

### 2.3 Single‐Device Data Modulation: Optical Logic Gate and Dual‐Channel Parallel Data Transmission

**Figure 4:**
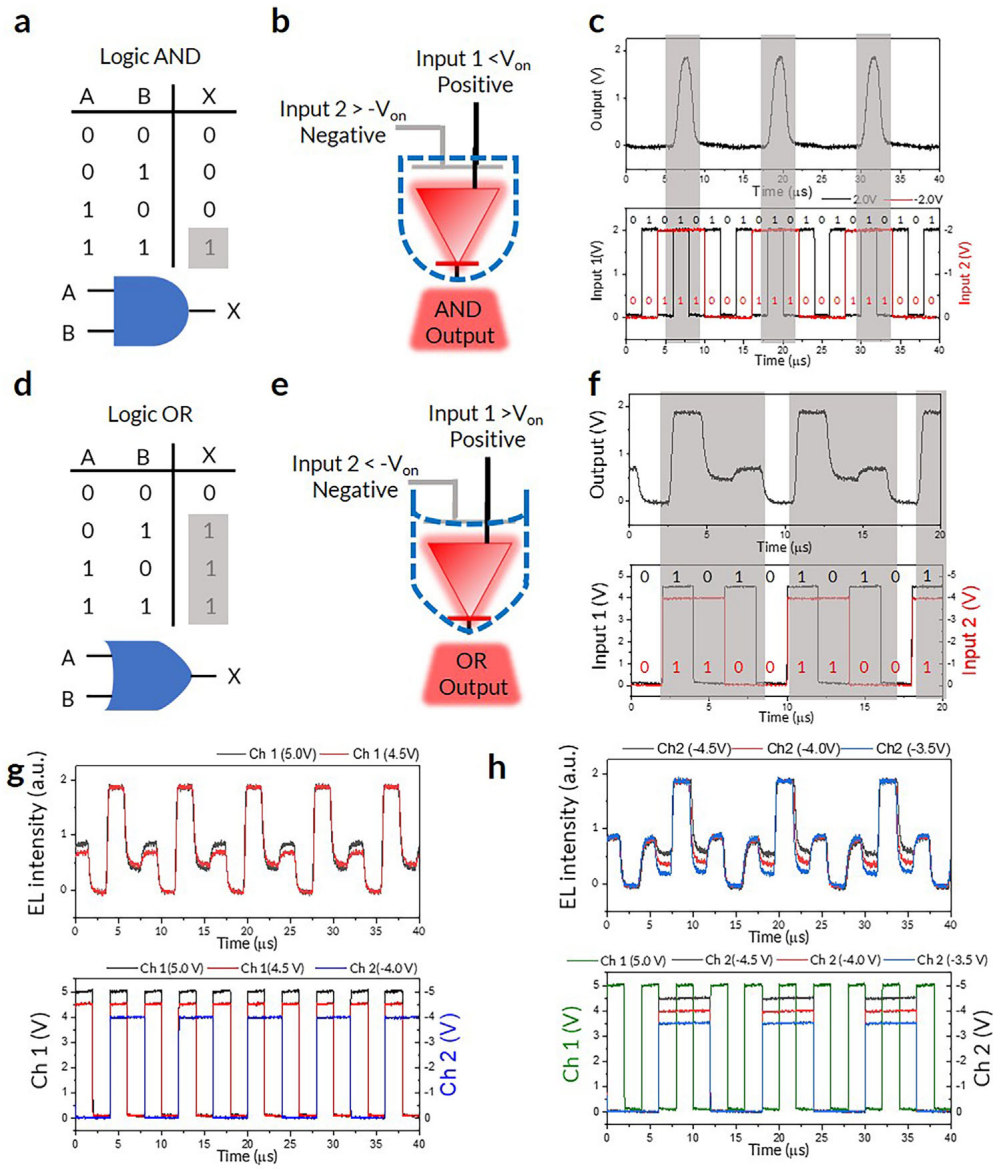
Single‐Device Data Modulation and Dual‐Channel Data Transmission. a) Truth table of logic AND. b), Operating condition of input data for AND output by PeLET. c), AND gate behavior, shown in gray boxes, when both inputs are in state = 1. d), Truth table of logic OR. e), Operating condition of input data for OR output by PeLET. f), OR gate behavior, shown in gray boxes, when either one or both inputs are in state = 1. Input 1 represents data input at the LED unit, and Input 2 represents input at the capacitor unit. g,h), Data output for dual‐channel data transmission with multi‐value EL levels; four different levels represent the different input circumstances in the truth table of logic OR. Each level is easily modulated via the applied bias at each channel. g), Mid‐level modulation through channel 1 (LED unit). h), Mid‐level modulation through channel 2 (capacitor unit).

The additional terminal of the PeLET expands its degrees of freedom for pulse signal modulation.^[^ ^7^ ^]^ Both the LED and capacitor terminals modify the EL intensity based on the intensity and period of the pulse (Figure 3). As both units transmit pulse data, each unit can be utilized as a separate input channel for an optical logic gate. Logic gates are the fundamental building blocks of digital circuits, by which digital systems perform logical functions.^[^ ^18^ ^]^ They are crucial for data processing, decision‐making, and complex calculations in electronic devices based on binary inputs.^[^ ^18^ ^]^ In PeLETs, binary electrical inputs A and B, one for each unit, are combined and translated into an optical output X (**Figure** **4**). This parallel data transmission through dual channels within a single PeLET overcomes the inherent speed limitations of conventional VLC devices.

To manipulate the binary electrical inputs, we established a binary‐input modulation program and setup (Figure S14, Supporting Information). Using a single PeLET device, we successfully implemented AND, OR, and Negative Implication (NIMPLY) logic gates (Figure 4a–f, and Figure S15, respectively, Supporting Information). Whereas traditional logic‐gate implementation would require at least six devices each for the AND and OR logic gates (Figure S16, Supporting Information), the PeLETs can modulate binary inputs to an optical output within a single device by controlling the input bias at each terminal (Figure 4f; Figure S17, Supporting Information).

For the OR logic gate, the manipulation of binary input data produces multi‐valued (here, three levels) EL intensities, which can be leveraged in VLC applications for dual‐channel data transmission. The transmitted data can be deconvoluted using a multi‐valued logic (MVL) algorithm.^[^ ^50^, ^51^ ^]^ The multilevel EL intensities, used to define the MVL truth table, can be modified by adjusting the voltage level of the pulse at each terminal (Figure 4g,h). The mid‐level intensities were successfully manipulated using both the capacitor and LED units. By varying the pulse ratio and duration, we measured pulse resolution in the PeLETs (Figure S17, Supporting Information). When a 2‐MHz pulse (pulse period of 0.5 µs) was applied to either the LED or capacitor terminal, the multi‐valued EL intensities became indistinguishable. The highest operating frequency condition for distinguishable multi‐value levels was achieved with a combination of 1 MHz at the LED and 0.5 MHz at the capacitor. This dual‐channel approach within a single PeLET device significantly enhances its data transmission capabilities by enabling the concurrent modulation and transmission of two independent data signals. The PeLET architecture offers the advantage of doubling the data throughput compared with those of traditional single‐channel devices while maintaining the LED configuration. However, the indistinguishability of the EL intensities at higher frequencies (≥2 MHz) suggests the need of further optimization of the pulse duration and capacitor layer to ensure signal accuracy. Addressing these challenges through additional research will be essential to enhancing the performance and reliability of PeLET‐based communication systems for advanced high‐speed VLC applications.

### 2.4 On‐Device Data Encryption Through Adjustable Interference Signals

Dual‐channel communications, particularly those utilizing the OR gate, provide a significant advantage in overcoming speed limitations through parallel data transmission. Moreover, additional channels provide new opportunities for data encryption during information transmission. The implementation of an interference layer introduces a novel concept for enhancing data security, by which noise or extra signals are added to distort the data and make them difficult to interpret. This ensures that, even in case of interception, the data cannot be deciphered without appropriate decoding keys, thus reducing the risk of unauthorized access and data breaches.

**Figure 5:**
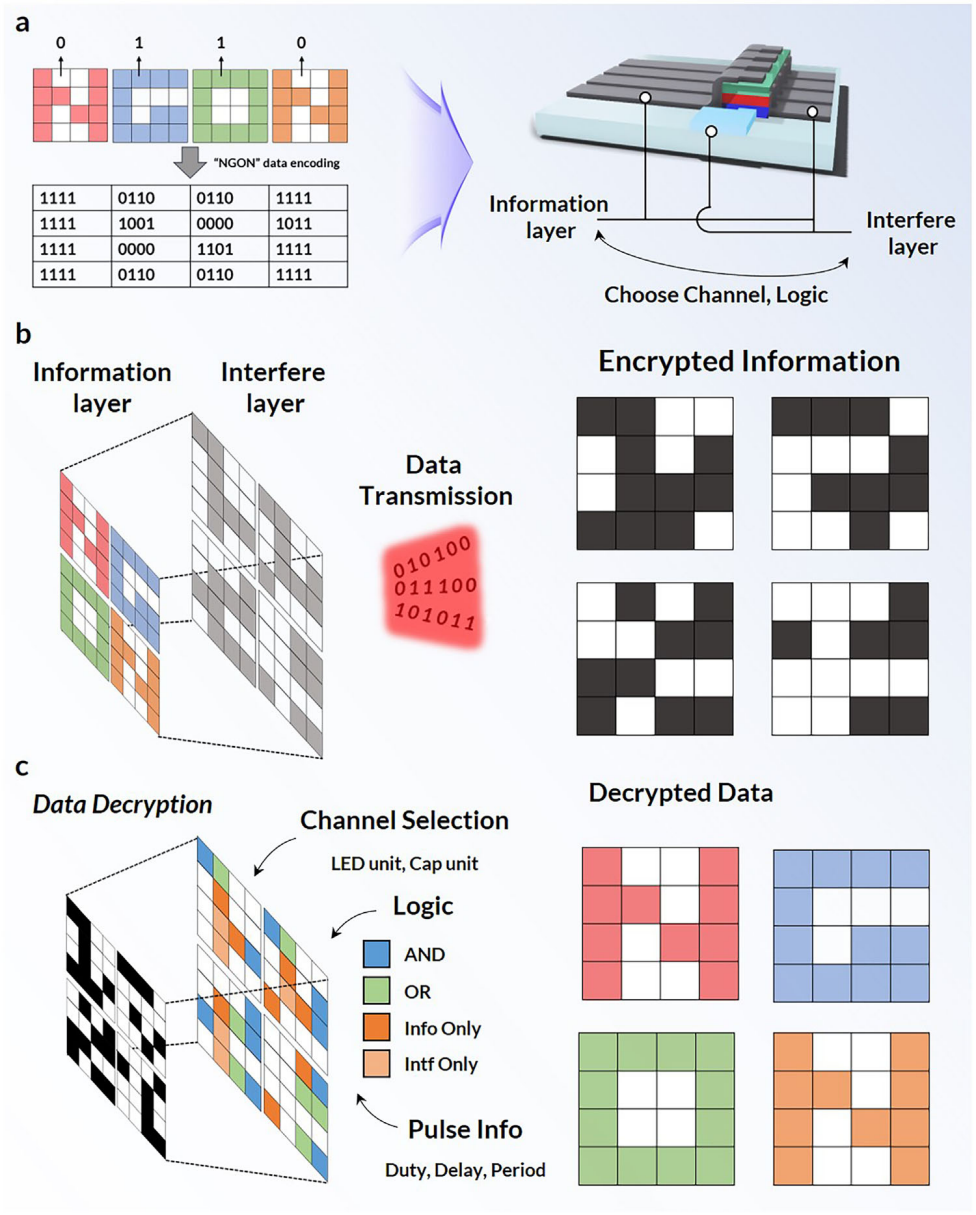
On‐Device Data Encryption. a), Encoded data for the word “NGON” (left) and scheme of on‐device data encryption (right), wherein the information layer and interference layer are switchable. b), Original word “NGON” projected through the interference layer and scrambled into encrypted information. c), Schematic for data decryption using the proper key. Key to unscramble the original data includes channel selection, identification of information layer (LED or capacitor unit), logic gate used, and pulse information.

By leveraging an additional channel for encryption, data transmitted via PeLET devices can be encoded with interference patterns that obscure the original signal. To demonstrate this concept of “on‐device data encryption,” we transmitted the word “NGON” through the PeLETs. The data were encoded as shown in **Figure** **5**a. The versatility of the PeLET architecture allows users to designate one channel as the interference layer and another as the information layer, offering flexibility for encryption schemes.

Furthermore, the variety of logic‐gate configurations provides numerous encryption possibilities. For example, the NIMPLY gate produces an output only if one of the binary inputs is in the ON state. In the PeLET encryption mechanism, this gate can be further configured to function as either “Info Only” or “Intf Only”, with both logics easily modulated by varying the pulse phase and voltage (Figure S12, Supporting Information). By selecting channels for the interference and information layers and configuring the logic gates accordingly, users can create highly diverse and robust encryption schemes.

In our encryption demonstration, the “NGON” data were encoded and encrypted by adjusting the interference layer using various logic gates and channel selections (Figure 5b; Figure S18, Supporting Information). As shown in Figure 5b, the output data were encrypted in an arbitrary pattern, making them unrelated to the original information. The encryption facilitated by the interference layer remains resistant to unauthorized access unless a correct decryption key is provided. This encryption system, referred to as Communications‐Electronics Operating Instructions (CEOI), is widely used in various applications, such as in the military. To decrypt and reconstruct the original data, several parameters must be known: the channel (LED or capacitor unit) selected as the information layer, the logic gate used, and details regarding the pulse durations for the interference signals. The use of CEOI to decrypt the encrypted data is shown in Figure S19 (Supporting Information), where the selected information layer is highlighted in gray, and each logic‐gate configuration is represented by different colors as in Figure 5c. To demonstrate the versatility of the encryption process, we explored various combinations of channel selection, logic gates, and pulse variation.

## 3 Conclusion

In this work, we present the PeLET architecture as a transformative solution to the limitations of conventional two‐terminal devices. The PeLET integrates vertically stacked capacitors and LED units, electrically connected by a permeable electrode. The pinholes in the permeable electrode were formed by utilizing the perforated morphology of zinc magnesium oxide ETL. The PeLET achieved a maximum EQE of 17.4% and a maximum luminance exceeding 29,000 cd m^−2^. Beyond these performance metrics, the PeLET device architecture suggests an alternative platform for VLC by integrating dual functionalities: optical logic gating, and on‐device data encryption. We observed two distinct TrEL behaviors depending on whether pulsed operations were applied to the capacitor or LED units and analyzed these behaviors in relation to E‐field penetration through the pinholes and RC characteristics. With precise control of the TrEL parameters, we demonstrated that optical logic gating, such as AND, OR, and NIMPLY operations, is achievable in PeLETs. This capability enables the simultaneous transmission of dual data streams within a single device. Significantly, PeLETs introduce a novel approach to data security by implementing an on‐device CEOI encryption mechanism that encodes optical information using interference layers. The scrambling of transmitted data enhances security by requiring specific configurations—encompassing pulse parameters, logic gate assignments, and terminal designations—for decryption. This encryption strategy can be further amplified through synergy with software‐based cryptographic methods, enhancing both flexibility and complexity in encryption protocols. This work positions the PeLET as a promising foundation for future VLC advancements, merging high‐efficiency light emission, dual‐channel communication, and robust data security within a single‐device architecture. Beyond traditional applications in display‐oriented VLETs (Supplementary Table S1), our work extends the functionality of vertical three‐terminal structures into the domain of optical communication by implemetning dual‐modulation capabilities, thereby broadening their potentional toward high‐speed and secure VLC systems.

## 4 Experimental Section

### Materials

Colloidal red InP quantum dots (QDs) (20 mg mL^−1^, dispersed in octane) were purchased from Uniam. Poly(3,4‐ethylenedioxythiophene) polystyrene sulfonate (PEDOT:PSS) (AI4083) was purchased from Hereus. Poly[(9,9‐dioctylfluorenyl‐2,7‐diyl)‐co‐(4,4‐(N‐(4‐sec‐butylphenyl)diphenylamine)] (TFB) was purchased from LumTec. Lithium fluoride (LiF) was supplied by Foosung Co., Ltd. Ethanol, dimethyl sulfoxide (DMSO), ethyl acetate, zinc acetate dihydrate, and magnesium acetate pentahydrate were purchased from Sigma‐Aldrich. Tetramethylammonium hydroxide (TMAH) was purchased from Thermo Fisher.

### Zn_87.5_Mg_12.5_O Nanoparticle Synthesis

Zinc acetate dihydrate (ZnAc, 0.5765 g) and magnesium acetate pentahydrate (MgAc, 0.0805 g) were dissolved in 30 mL of DMSO with vigorous stirring. TMAH (0.9062 g) was dissolved in 10 mL of ethanol. After 30 min, the TMAH solution was injected rapidly into the ZnAc and MgAc solution. After the solution became transparent, the ZnMgO solution was washed with ethyl acetate, centrifuged for 5 min at 4500 rpm, and redispersed in ethanol.

### Materials Characterization

The XPS (Al kα line1486.6 eV) and UPS spectra were measured using Axis‐Supra of Kratos. Images of the morphology were obtained using a field‐emission scanning electron microscope (SU‐5000, Hitachi).

### Fabrication of PeLET

Indium tin oxide (ITO)‐patterned glasses were sonicated in acetone and 2‐propanol for 15 min each. The mixture was then boiled in 2‐propanol for 30 min. The ITO substrates were subjected to an UV–ozone treatment to achieve a hydrophilic surface before spin‐coating with PEDOT:PSS. The PEDOT:PSS was spin‐coated onto the substrate at 4500 rpm for 30 s with 3 s of the pre‐spin process at 500 rpm, then annealed at 120 °C for 30 min. TFB was then coated onto the material at 3000 rpm for 40 s and annealed at 120 °C for 10 min. Prior to the InP QD coating process, the substrates were placed in a nitrogen‐filled glovebox. For the InP QD coating, the pre‐spin and solvent evaporation were performed for 2 s at 500 rpm and 30 s at 3000 rpm, respectively. After the InP QD coating process, ZnMgO nanoparticles were coated onto the material at 4500 rpm for 30 s and annealed at 150 °C for 30 min. Immediately after the coating process, the samples were transferred to a vacuum chamber (<5 × 10^−6^ Torr), and a permeable cathode of Al was deposited to 25 nm at a deposition rate of 0.2A s^−1^ and substrate rotational speed of 4 rpm. After the formation of the permeable cathode, the dielectric LiF (80 nm at 0.5A s^−1^) and Al electrode (1 A/s for 20 nm, then boosted to 2.5A/s for 80 nm) were deposited. For smooth contact between the electrode and each layer, the devices were annealed at 150 °C for 30 min. All device characterizations were performed after the device had been cooled down.

### Device Characterization

The external quantum efficiency (EQE) measurement system (M6000) was integrated with two Keithley 2400 source meter units and a Konica Minolta CS‐2000 spectroradiometer (McScience). The I‐V sweeps (Cap unit sweep and LED unit sweep)were measured using two Keithley 2400 source meter units and the open‐source software SweepMe.

### Three‐Terminal Transient Electroluminescence and Single‐Device Data Modulation

The transient EL was measured using an integrated system comprising a function generator (AFG31022), oscilloscope (DPO4054), source meter (Keithley 2400), and PMT (Figure 4a). In the PMT‐based measurement setup, the PMT output current is linearly proportional to the incident optical power at a given wavelength with radiant sensitivity value *S* (mA/W). Thus the – 3dB bandwidth was calculated by the given equation below:

(1) $$-3dB=10log\;\frac{P}{P_{ref}}=10log\;\frac{\left(I/S\right)}{\left(I_{ref}/S\right)}=10log\;\frac{I}{I_{ref}}$$

where *P* and *I* is output optical power and response current of PMT respectively. In addition, the single‐device data modulation, including the optical logic gate and on‐device data encryption, was measured by two separate pulse modulations using a synchronized pulse input from the function generator. This equipment was controlled using the Python library PyVisa.

## Conflict of Interest

The authors declare no conflict of interest.

## Author Contributions

S.Shin developed the ideas, performed the most experiments, and wrote the manuscript. H.Lee supported synthesizing ZnMgO nanoparticles and observing TEM. W.Lee performed resistance characterization of electrodes. S.Lee contributed to the SEM measurements. K.G.Lim and H.Cho supervised the entire project and revised the manuscript.

## Supporting information



Supporting Information

## Data Availability

The data that support the findings of this study are available from the corresponding author upon reasonable request.

## References

[1] Y.‐H. Won , O. Cho , T. Kim , D. Y. Chung , T. Kim , H. Chung , H. Jang , J. Lee , D. Kim , E. Jang , Nature 2019, 575, 634. 31776489 10.1038/s41586-019-1771-5

[2] , J. S. Kim , J. M. Heo , G. S. Park , S. J. Woo , C. Cho , H. J. Yun , D. H. Kim , Nature 2022, 611, 688. 36352223 10.1038/s41586-022-05304-w

[3] A. Mischok , S. Hillebrandt , S. Kwon , M. C. Gather , Nat. Photonics 2023, 17, 393.

[4] , J. J. Yoo , G. Seo , M. R. Chua , T. G. Park , Y. Lu , F. Rotermund , Y. K. Kim , Nature, 590, 587. 33627807 10.1038/s41586-021-03285-w

[5] A. J. J. M. van Breemen , R. Ollearo , S. Shanmugam , B. Peeters , L. C. J. M. Peters , R. L. van de Ketterij , I. Katsouras , H. B. Akkerman , C. H. Frijters , F. Di Giacomo , S. Veenstra , R. Andriessen , R. A. J. Janssen , E. A. Meulenkamp , G. H. Gelinck , Nat. Electron. 2021, 4, 818.

[6] A. M. Najarian , M. Vafaie , A. Johnston , T. Zhu , M. Wei , M. I. Saidaminov , Y. Hou , S. Hoogland , F. Pelayo García de Arquer , E. H. Sargent , Nat. Electron. 2022, 5, 511.

[7] M. H. Memon , H. Yu , Y. Luo , Y. Kang , W. Chen , D. Li , D. Luo , S. Xiao , C. Zuo , C. Gong , C. Shen , L. Fu , B. S. Ooi , S. Liu , H. Sun , Nat. Electron. 2024, 7, 279.

[8] Z. Wu , Y. Liu , E. Guo , G. Darbandy , S. J. Wang , R. Hübner , A. Kloes , H. Kleemann , K. Leo , Nat. Mater. 2021, 20, 1007. 33649562 10.1038/s41563-021-00937-0

[9] M. A. McCarthy , B. Liu , E. P. Donoghue , I. Kravchenko , D. Y. Kim , F. So , A. G. Rinzler , Science 2011, 332, 570. 21527708 10.1126/science.1203052

[10] A. J. Taal , I. Uguz , S. Hillebrandt , C.‐K. Moon , V. Andino‐Pavlovsky , J. Choi , C. Keum , K. Deisseroth , M. C. Gather , K. L. Shepard , Nat. Electron. 2023, 6, 669.

[11] D. Geng , K. Wang , L. Li , K. Myny , A. Nathan , J. Jang , Y. Kuo , M. Liu , Nat. Electron. 2023, 6, 963.

[12] H. Yu , Z. Dong , J. Guo , D. Kim , F. So , ACS Appl. Mater. Interfaces 2016, 8, 10430. 27082815 10.1021/acsami.6b00182

[13] X. Wu , C. Gao , Q. Chen , Y. Yan , G. Zhang , T. Guo , H. Chen , Nat. Commun. 2023, 14, 1579. 36949063 10.1038/s41467-023-37174-9 PMC10033512

[14] H. R. Sim , S. Lee , J. Lee , S. Z. Hassan , G.‐H. Nam , C. So , K. M. Sim , D. S. Chung , ACS Nano 2023, 17, 24374. 38039187 10.1021/acsnano.3c10663

[15] C. Gao , H. Yang , E. Li , Y. Yan , L. He , H. Chen , Z. Lin , T. Guo , ACS Photonics 2021, 8, 3094.

[16] E. Li , X. Wu , Q. Chen , S. Wu , L. He , R. Yu , Y. Hu , H. Chen , T. Guo , Nano Energy 2021, 85, 106010.

[17] F. Dollinger , K. G. Lim , Y. Li , E. Guo , P. Formánek , R. Hübner , A. Fischer , H. Kleemann , K. Leo , Adv. Mater. 2019, 31, 1900917. 10.1002/adma.201900917 30920705

[18] E. Guo , Z. Wu , G. Darbandy , S. Xing , S. J. Wang , A. Tahn , M. Göbel , A. Kloes , K. Leo , H. Kleemann , Nat. Commun. 2020, 11, 4725. 32948770 10.1038/s41467-020-18576-5 PMC7501854

[19] A. Ren , H. Wang , W. Zhang , J. Wu , Z. Wang , R. V. Penty , I. H. White , Nat. Electron. 2021, 4, 559.

[20] S. Dang , O. Amin , B. Shihada , M. S. Alouini , Nat. Electron. 2020, 3, 20.

[21] D. Karunatilaka , F. Zafar , V. Kalavally , R. Parthiban , IEEE Communications Surveys & Tutorials 2015, 17, 1649.

[22] H. Elgala , R. Mesleh , H. Haas , IEEE Communications Magazine 2011, 49, 56.

[23] Y. K. Cheong , X. W. Ng , W. Y. Chung , IEEE Sens. J. 2013, 13, 3347.

[24] J. An , N. Q. Pham , W. Y. Chung , Opt. Commun. 2017, 405, 107.

[25] Z. Wei , L. Wang , Z. Liu , C. Zhang , C.‐J. Chen , M.‐C. Wu , Y. Yang , C. Yu , L. Wang , H. Y. Fu , ACS Photonics 2022, 9, 2354.

[26] C. Du , C. Jiang , P. Zuo , X. Huang , X. Pu , Z. Zhao , Y. Zhou , L. Li , H. Chen , W. Hu , Z. L. Wang , Small 2015, 11, 6071. 26450795 10.1002/smll.201502170

[27] F.‐H. Hsiao , W. C. Miao , T.‐Y. Lee , Y.‐H. Pai , Y.‐Y. Hung , D. Iida , C. L. Lin , C. W. Chow , G.‐R. Lin , K. Ohkawa , H. C. Kuo , Y.‐H. Hong , Sci. Rep. 2024, 14, 7018. 38528020 10.1038/s41598-024-57132-9 PMC10963728

[28] L. Hu , J. Choi , S. Hwangbo , D.‐H. Kwon , B. Jang , S. Ji , J. H. Kim , S. K. Han , J. H. Ahn , npj Flexible Electron. 2022, 6, 1.

[29] K. Yoshida , P. P. Manousiadis , R. Bian , Z. Chen , C. Murawski , M. C. Gather , H. Haas , G. A. Turnbull , I. D. W. Samuel , Nat. Commun. 2020, 11, 1171. 32127529 10.1038/s41467-020-14880-2 PMC7054290

[30] A. Minotto , P. A. Haigh , Ł. G. Łukasiewicz , E. Lunedei , D. T. Gryko , I. Darwazeh , F. Cacialli , Light: Sci. Appl. 2020, 9, 70. 32351694 10.1038/s41377-020-0314-z PMC7183573

[31] D. Kim , H. J. Park , S. H. Jung , W. J. Pyo , S. Z. Hassan , H. R. Sim , J. H. Lee , D. W. Jee , D. S. Chung , Adv. Mater. 2024, 36, 2309416. 10.1002/adma.202309416 37856894

[32] Y. Wang , H. Chen , W. Jiang , X. Li , X. Chen , X. Meng , P. Tian , B. Sun , Optics and Lasers in Engineering 2020, 134, 106290.

[33] L. Bai , Z. Xie , W. Wang , C. Yuan , Y. Zhao , Z. Mu , Q. Zhong , Z. Gu , ACS Nano 2014, 8, 11094. 25300045 10.1021/nn504659p

[34] F. Dong , W. Chu , Adv. Mater. 2019, 31, 1804921. 10.1002/adma.201804921 30556627

[35] S. Liu , X. Liu , X. Zhu , J. Yin , J. Bao , ACS Nano 2023, 17, 21349. 37883096 10.1021/acsnano.3c06050

[36] T. Sarkar , K. Selvakumar , L. Motiei , D. Margulies , Nat. Commun. 2016, 7, 11374. 27138465 10.1038/ncomms11374 PMC4857388

[37] X. Fang , H. Ren , M. Gu , Nat. Photonics 2020, 14, 102.

[38] S. A. Maier , Plasmonics: Fundamentals and Applications, Springer US, New York, NY 2007.

[39] H. Yu , J. H. Kim , W. Chen , D. Kim , J. Guo , F. So , Adv. Funct. Mater. 2014, 24, 6056.

[40] M. R. O'Masta , E. C. Clough , J. H. Martin , Comput. Mater. Sci. 2021, 192, 110317.

[41] A. Karoui , ECS Trans. 2011, 41, 21.

[42] I. Dursun , C. Shen , M. R. Parida , J. Pan , S. P. Sarmah , D. Priante , N. Alyami , J. Liu , M. I. Saidaminov , M. S. Alias , A. L. Abdelhady , T. K. Ng , O. F. Mohammed , B. S. Ooi , O. M. Bakr , ACS Photonics 2016, 3, 1150.

[43] E. Jang , H. Jang , Chem. Rev. 2023, 123, 4663. 36795794 10.1021/acs.chemrev.2c00695

[44] S. Chen , W. Cao , T. Liu , S. W. Tsang , Y. Yang , X. Yan , L. Qian , Nat. Commun. 2019, 10, 765. 30770861 10.1038/s41467-019-08749-2 PMC6377672

[45] K. J Kim , T. Kim , Journal of Industrial and Engineering Chemistry 2023, 119, 598.

[46] L. P. Keating , H. Lee , S. P. Rogers , C. Huang , M. Shim , Nano Lett. 2022, 22, 9500. 36459088 10.1021/acs.nanolett.2c03564

[47] P. Yu , Q. Yuan , J. Zhao , H. Zhang , W. Ji , J. Phys. Chem. Lett. 2022, 13, 2878. 35333050 10.1021/acs.jpclett.2c00604

[48] H. M. Kim , D. Geng , J. Kim , E. Hwang , J. Jang , ACS Appl. Mater. Interfaces 2016, 8, 28727. 27696804 10.1021/acsami.6b10314

[49] H. H. Cho , S. Gorgon , H. C. Hung , J.‐Y. Huang , Y. R. Wu , F. Li , N. C. Greenham , E. W. Evans , R. H. Friend , Adv. Mater. 2023, 35, 2303666. 10.1002/adma.202303666 37684741

[50] E. Zaitseva , V. Levashenko , Journal of Applied Logic 2013, 11, 350.

[51] M. Andreev , S. Seo , K.‐S. Jung , J. H. Park , Adv. Mater. 2022, 34, 2108830. 10.1002/adma.202108830 35894513

