# Supplementary material for: High‐Efficiency Quantum Dot Permeable Electrode Light‐Emitting Triodes for Visible Light Communications and on‐Device Data Encryption
Source: Adv Mater. 2025 May 30;37(38):2503189. doi: 10.1002/adma.202503189 (PMC12464657; doi:10.1002/adma.202503189)
Supplement: Supplementary file 1 — Supporting Information [file ADMA-37-2503189-s001.docx]

**Supporting Information**

High-Efficiency Quantum Dot Permeable Electrode Light-Emitting Triodes for Visible Light Communications and On-Device Data Encryption

Seungmin Shin^1^, Hyungdoh Lee^1^, Wonbeom Lee^1^, Seungwoo Lee^2^, Kyung-geun Lim^3,^*, Himchan Cho^1,2,^*

*^1^Department of Materials Science and Engineering, Korea Advanced Institute of Science and Technology (KAIST), Daejeon, 34141, Republic of Korea*

*^2^Graduate School of Semiconductor Technology, School of Electrical Engineering, Korea Advanced Institute of Science and Technology (KAIST), Daejeon, 34141, Republic of Korea*

*^3^Korea Research Institute of Standards and Science (KRISS), Daejeon 34113, Republic of Korea*

**E-mail:** [**kglim@kriss.re.kr**](mailto:kglim@kriss.re.kr)**;** [**himchan@kaist.ac.kr**](mailto:himchan@kaist.ac.kr)

**Table of Contents**

**Figure S1.** Device architecture of conventional LEDs and PeLETs

**Figure S2.** Perforated morphology of permeable electrode.

**Figure S3.** Engineering Al electrode.

**Figure S4.** Engineering ZnMgO ETL to enhance efficiency

**Figure S5**. XPS, UPS and absorption properties.

**Figure S6**. Colloidal Stability by DMSO treatment.

**Figure S7.** Schematic of C–V measurement.

**Figure S8.** Current densities of LED and capacitor units.

**Figure S9.** Controllability of LED luminance via capacitor unit bias

**Figure S10.** 3-dimensional TrEL intensities.

**Figure S11.** Description of EL over shoot by Cap pulse.

**Figure S12.** Maximum TrEL intensities rising tendency.

**Figure S13.** -3dB on-off keying bandwidth.

**Figure S14.** Measurement setup for single-device data modulation.

**Figure S15.** NIMPLY logic circuit.

**Figure S16.** Structure of traditional logic circuit.

**Figure S17.** Dual-channel data transmission with various pulse period.

**Figure S18.** Raw data of encrypted data.

**Figure S19.** CEOI for decryption.

**Supplementary Note 1.** Pinhole formation by Bénard–Marangoni convection.

**Supplementary Note 2.** Analysis of capacitor current between PE and fully filled electrode.

Supplementary Note 3. Define the time constant for the TrEL measurement.

Supplementary Note 4. Supplementary discussion for VLC application

Supplementary Table 1. Overview of reported vertical three-terminal light-emitting devices and their key characteristics

Figure S1.


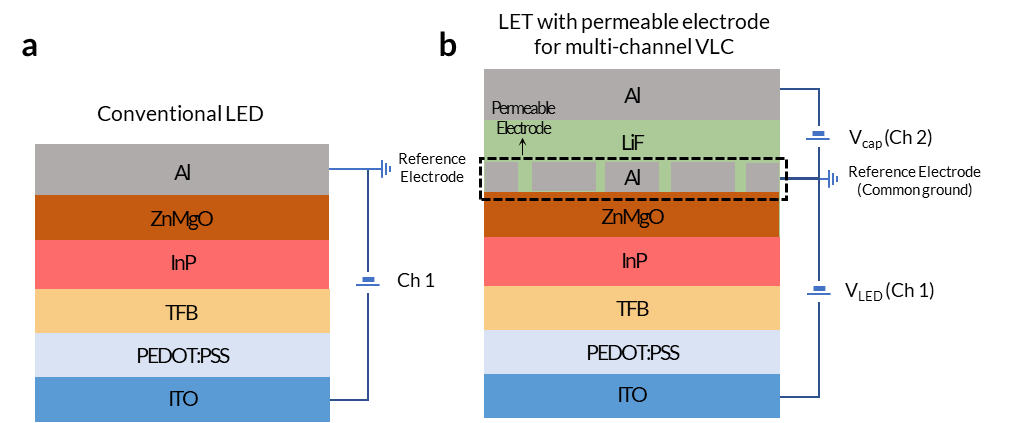


Figure S1 Device Architecture of Conventional LEDs and PeLETs. a,b Schematic device architecture and electrode configuration of conventional LEDs and PeLETs, respectively.

Figure S2.


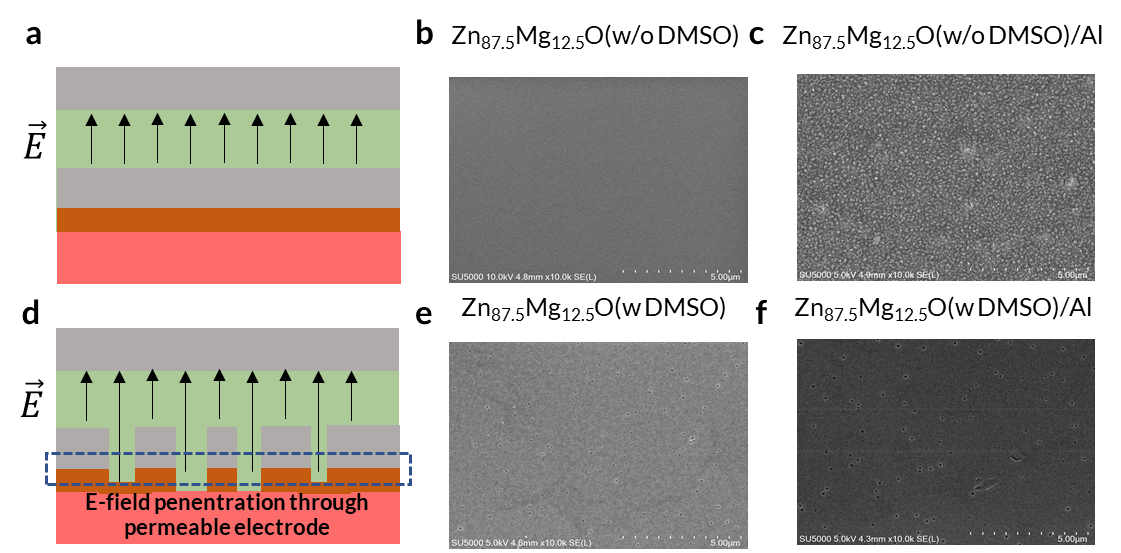


Figure S2 Perforated Morphology of Permeable Electrode. a, Schematic of electric field shielded by non-perforated electrode. b, c, SEM images of non-perforated ZnMgO and Al surfaces, respectively. d, Schematic of electric field penetration through permeable electrode. e, f, SEM images of perforated ZnMgO and Al surfaces, respectively.

Figure S3.


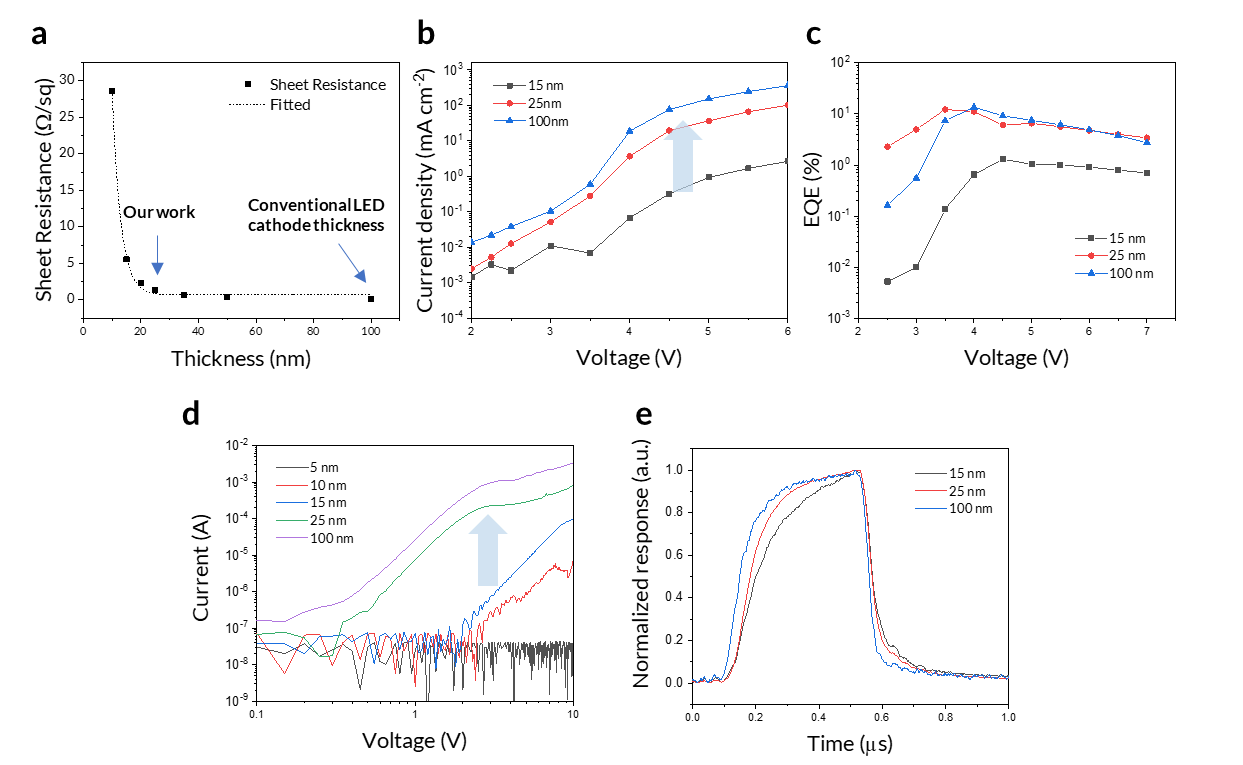


Figure S3. a. Sheet resistance varied by Al thickness.b, c. Current density and EQE varied by Al thickness, d. Current in electron only device (ITO/ZnMgO/InP/ZnMgO/Al) varied by Al thickness, e. Transient electroluminescence varied by Al thickness

Figure S4.


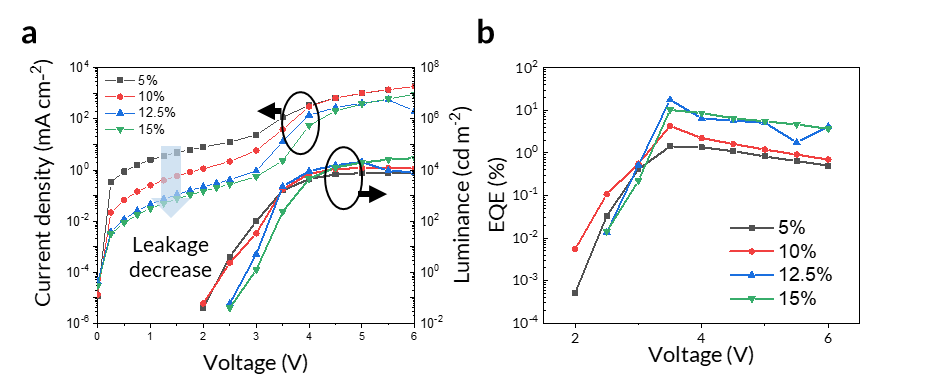


Figure S4. a. Current density(left) and Luminance(right) data varied by Mg composition in Zn_1-x_Mg_x_O ETL. b. EQE data varied by Mg composition

Figure S5.


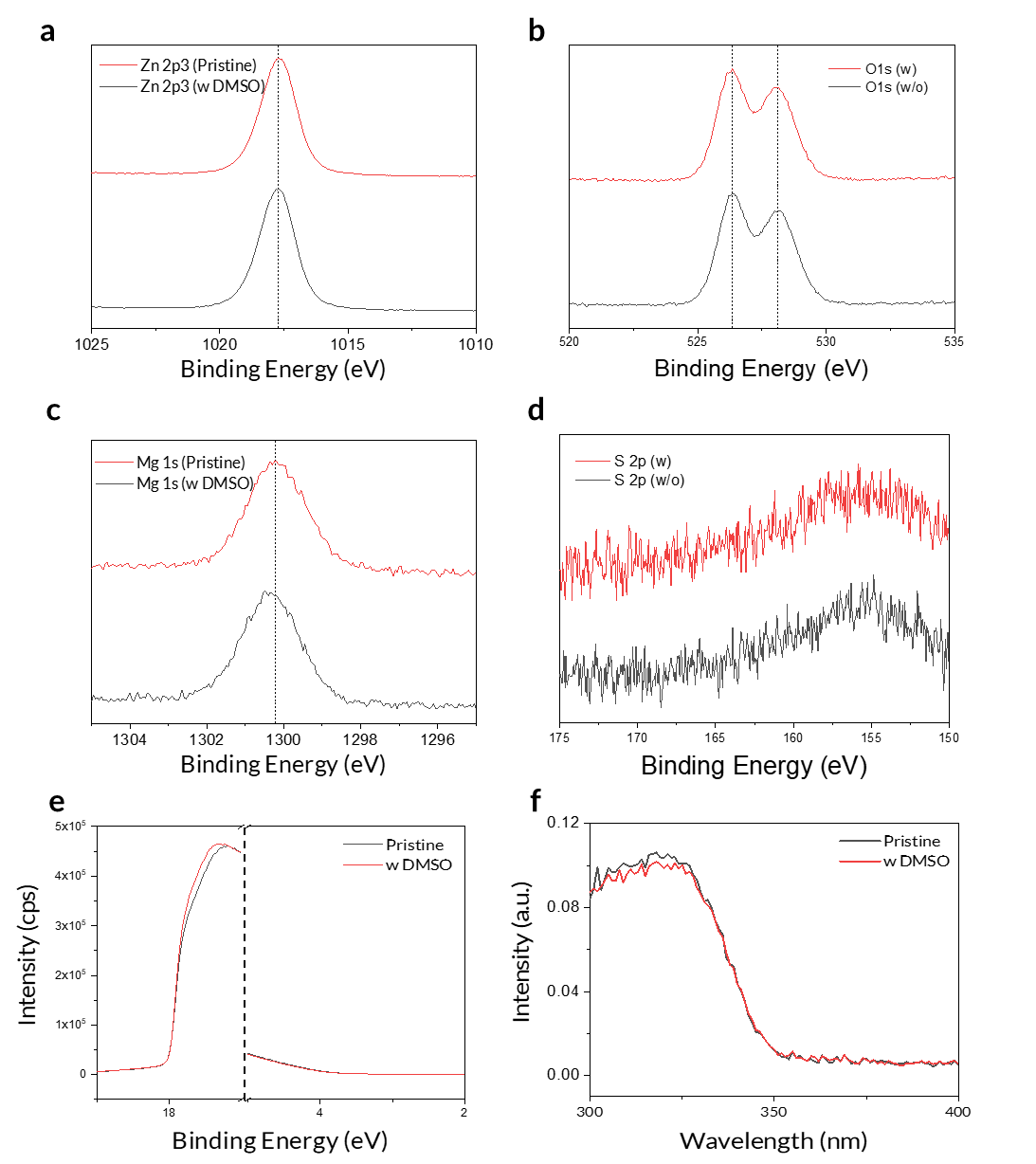


Figure S5 a, b, c, d, XPS spectra of Zn 2p3, O 1s, Mg 1s, S 2p, respectively. e, UPS spectra. f, Absorbance with and without DMSO treatment.

Figure S6.


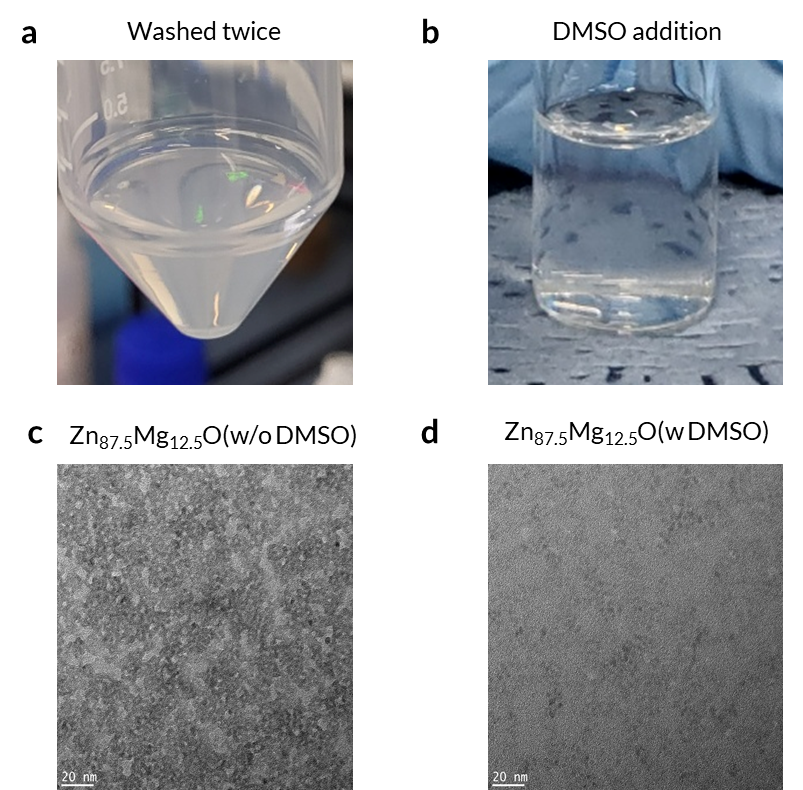


Figure S6 Colloidal Stability by DMSO Treatment. a, Turbid Zn_87.5_Mg_12.5_O solution after washing twice. b, Well-dispersed Zn_87.5_Mg_12.5_O solution after addition of DMSO. c, d, TEM images of Zn_87.5_Mg_12.5_O without and with DMSO, respectively.

Figure S7.


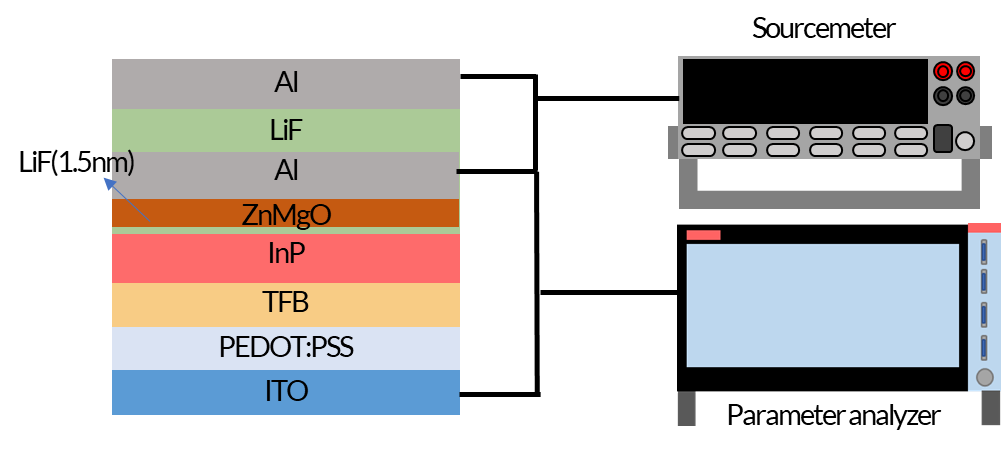


Figure S7. Capacitance–Voltage measurement with charge accumulation layer.

Figure S8.


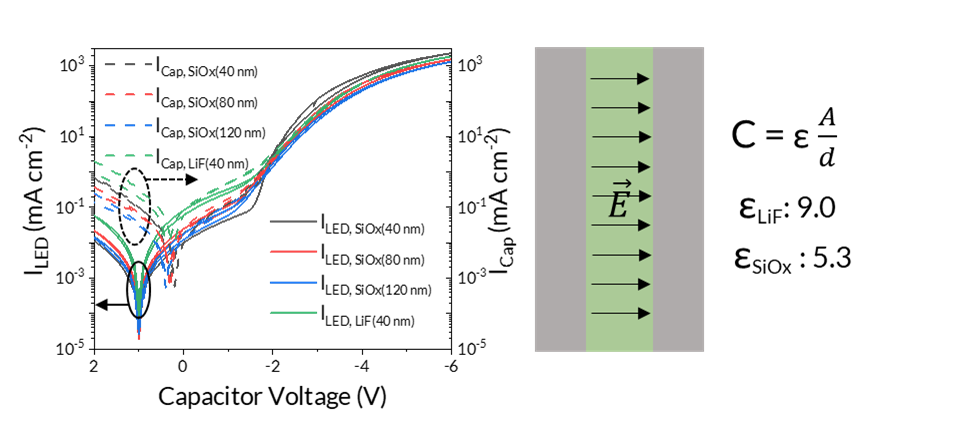


Figure S8. Current densities of LED and capacitor units vs. capacitor voltage (applied bias at capacitor unit), varied by dielectric layer and its thickness; solid and dashed lines represent current densities at LED and capacitor units, respectively. (left); schematic of parallel capacitor structure and its equation (right).

Figure S9.


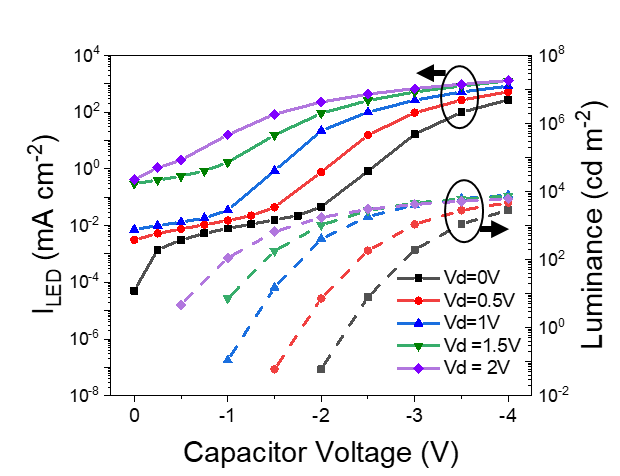


Figure S9 Controllability of LED luminance via capacitor unit bias

**Figure S10.**

**
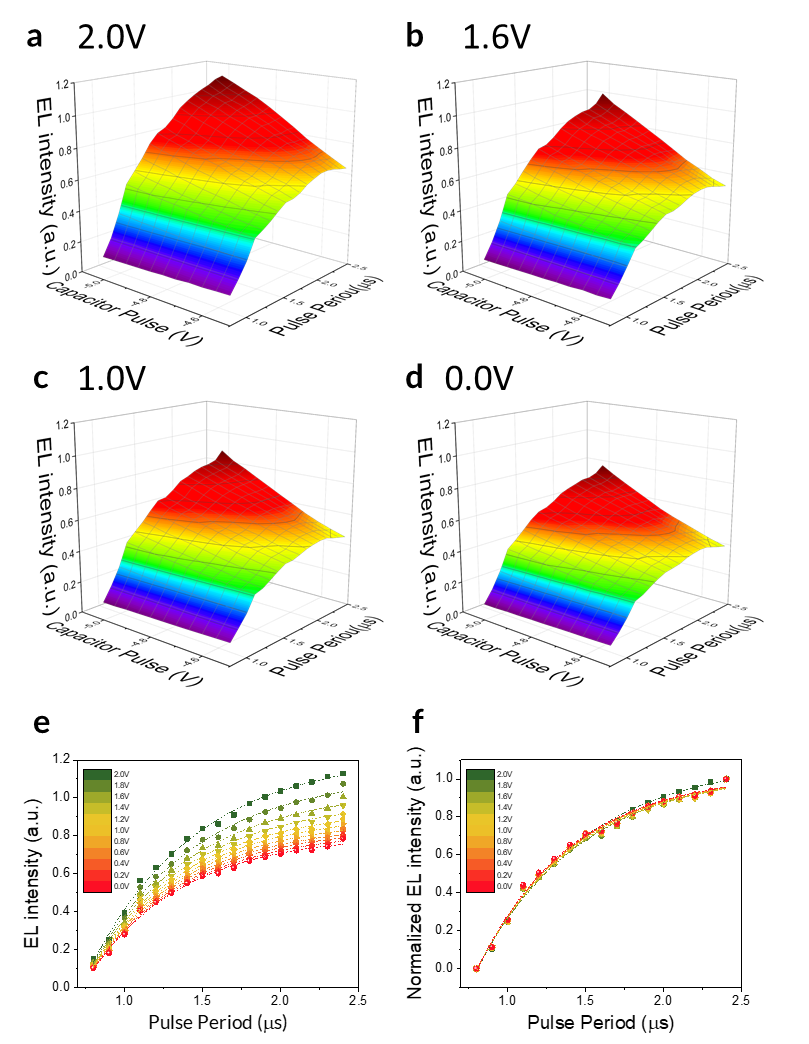
**

Figure S10 a, b, c, d, 3-dimensional EL intensity plots for different DC biases applied to LED unit. e, EL with respect to pulse period and DC bias applied to LED unit. f, Normalized EL with respect to pulse period and DC bias applied to LED unit.

**Figure S11.**

**
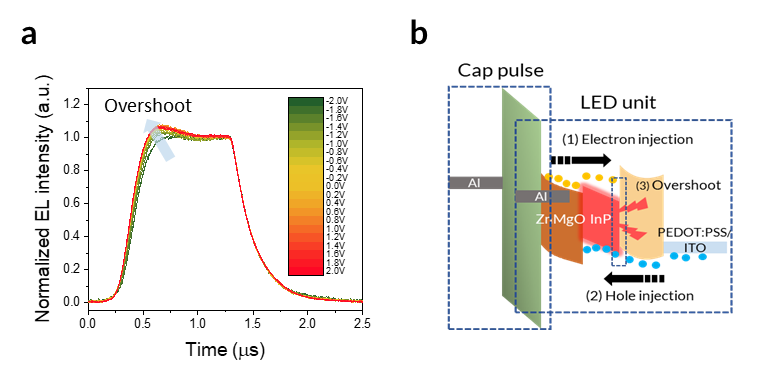
**

Figure S11 a, Transient EL overshoot with respect to DC bias at the LED unit (Cap pulse voltage = -5V). b, Schematics of electronic band structure of PeLETs describing transient EL overshoot mechanism.

Figure S12.


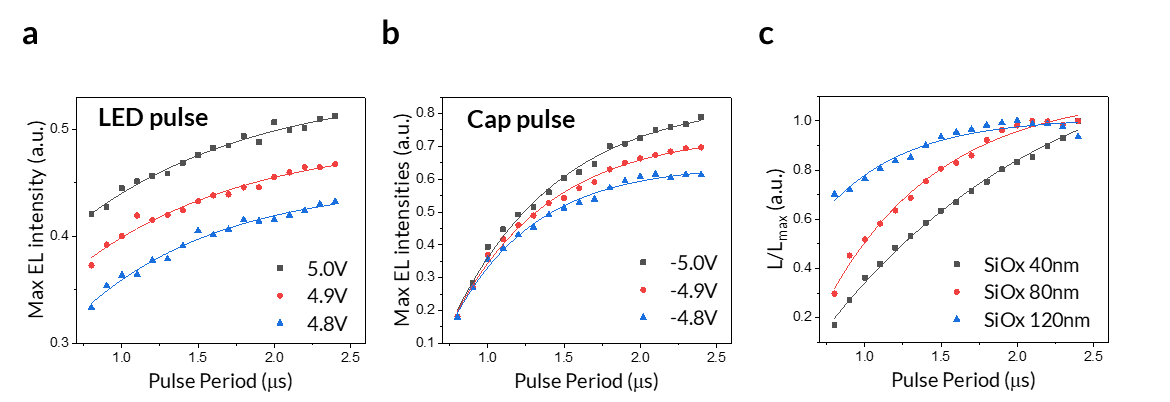


Figure S12 a, b, Maximum EL intensities exhibiting rising tendencies as pulse period is increased at both LED and capacitor units, respectively. c, RC tendency of EL intensity for −5.0 V applied bias at capacitor unit with respect to different thicknesses of dielectric layer.

**Figure S13.**

**
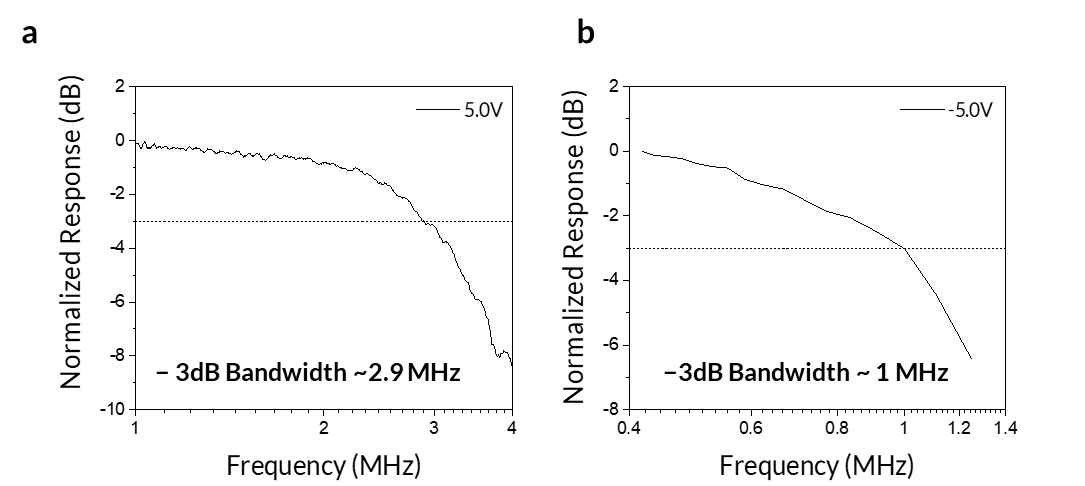
**

Figure S13 a, b −3 dB On-off keying bandwidth of LED-unit, and capacitor unit respectively

**Figure S14.**

**
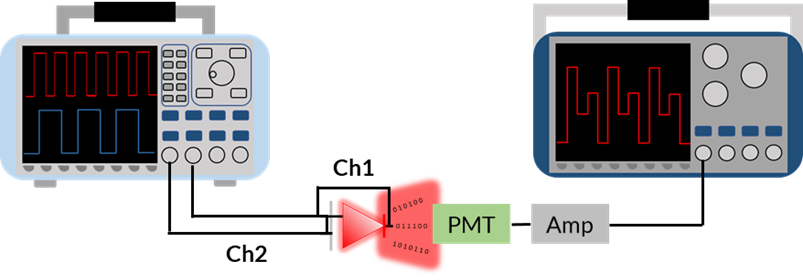
**

Figure S14. Measurement setup for single-device data modulation.

**Figure S15.**

**
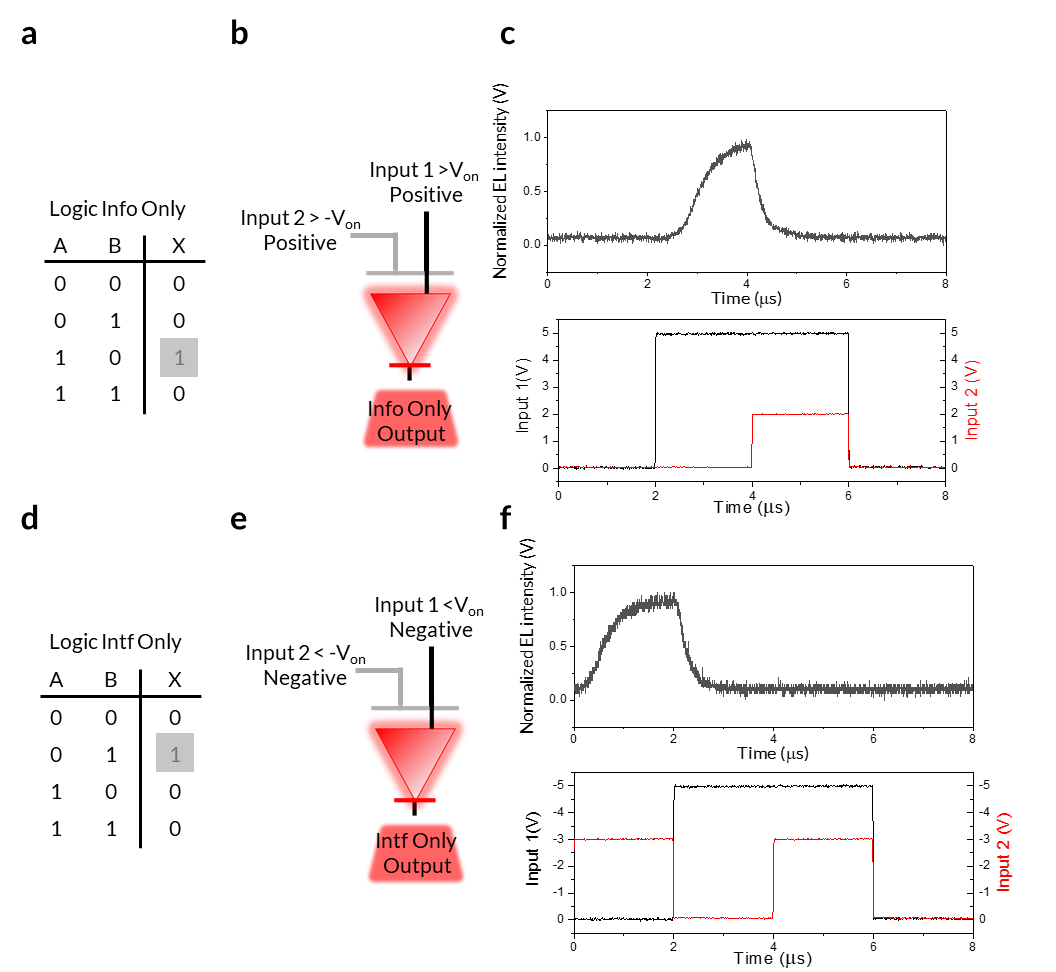
**

Figure S15 a, Truth table of logic “Info Only”. b, Operating condition of input data for “Info Only” output by PeLET. c, “Info Only” gate behavior when only input A is in state = 1. d, Truth table of logic “Intf Only”. e, Operating condition of input data for “Intf Only” output by PeLET. f, “Intf Only” gate behavior when only input B is in state = 1. Both “Info Only” and “Intf Only” are included in NIMPLY logic but, for further application of on-device data encryption, are recategorized into separate names for the logic gate.

**Figure S16.**

**
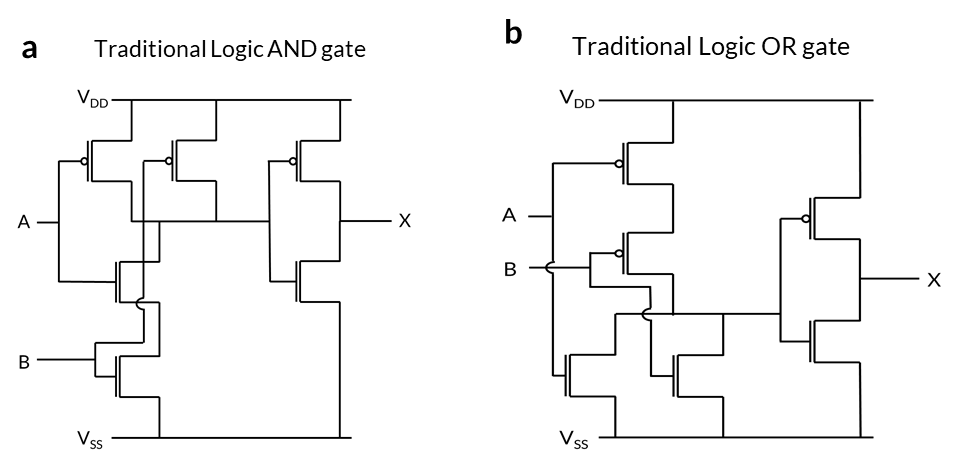
**

Figure S16 a, Structure of traditional AND logic gate. b, Structure of traditional OR logic gate.

**Figure S17.**

**
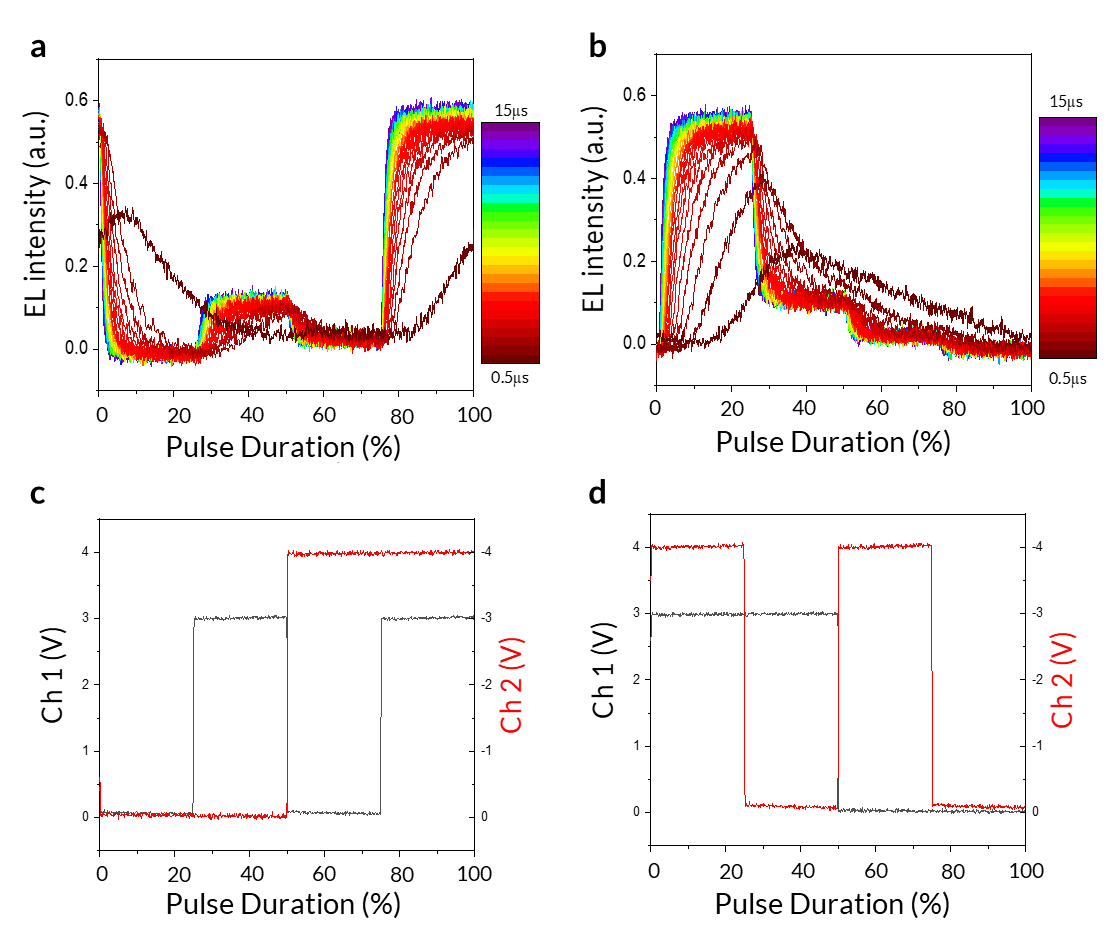
**

Figure S17 a, b, Dual-channel data transmission and multi-value tunability with respect to decreasing pulse period. c, d, Pulse information for a and b, respectively. Channel 1 represents data input at LED unit, and channel 2 represents data input at capacitor unit.

**Figure S18.**

**
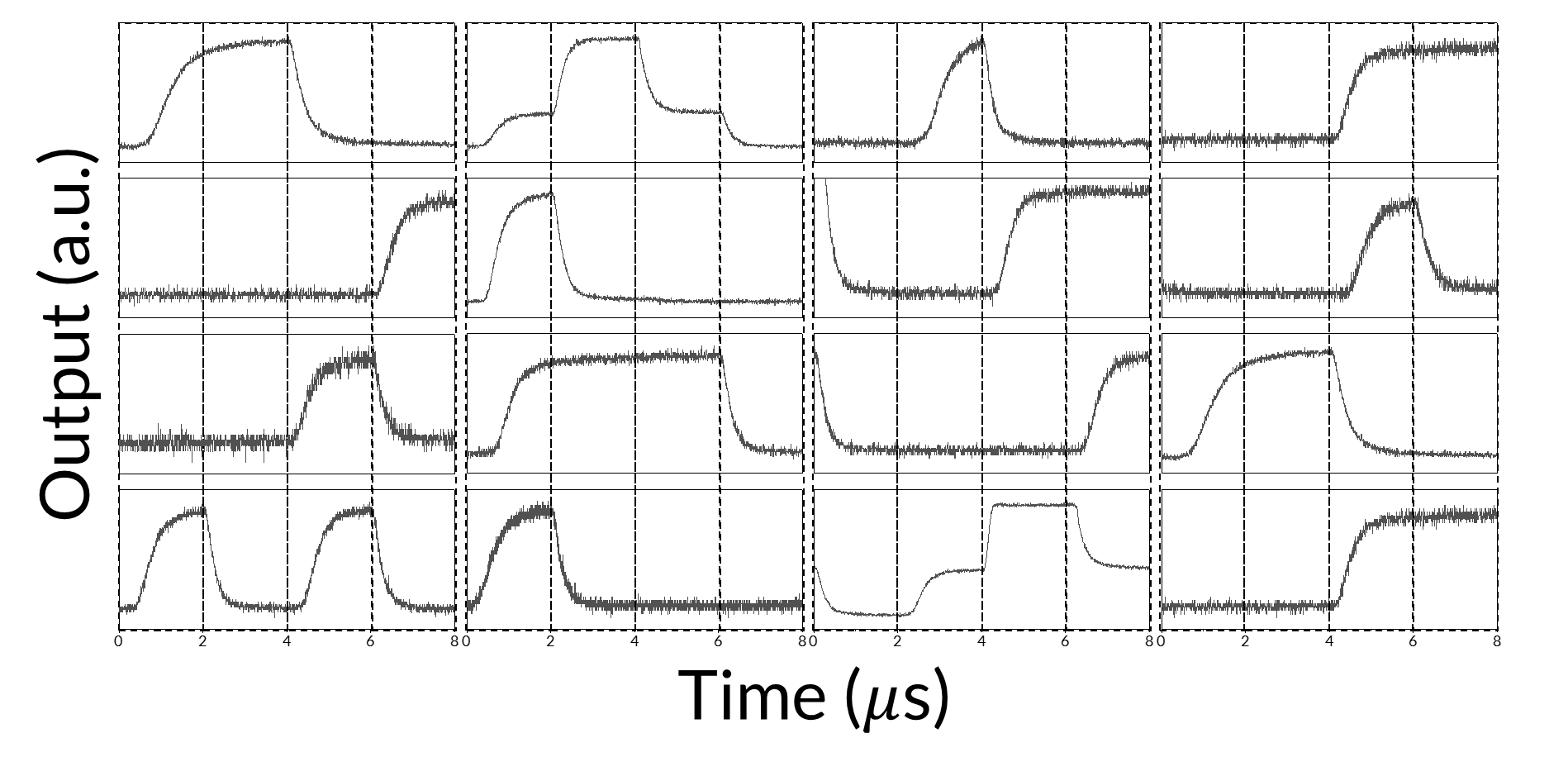
**

Figure S18. Raw data of encrypted “NGON”.

**Figure S19.**

**
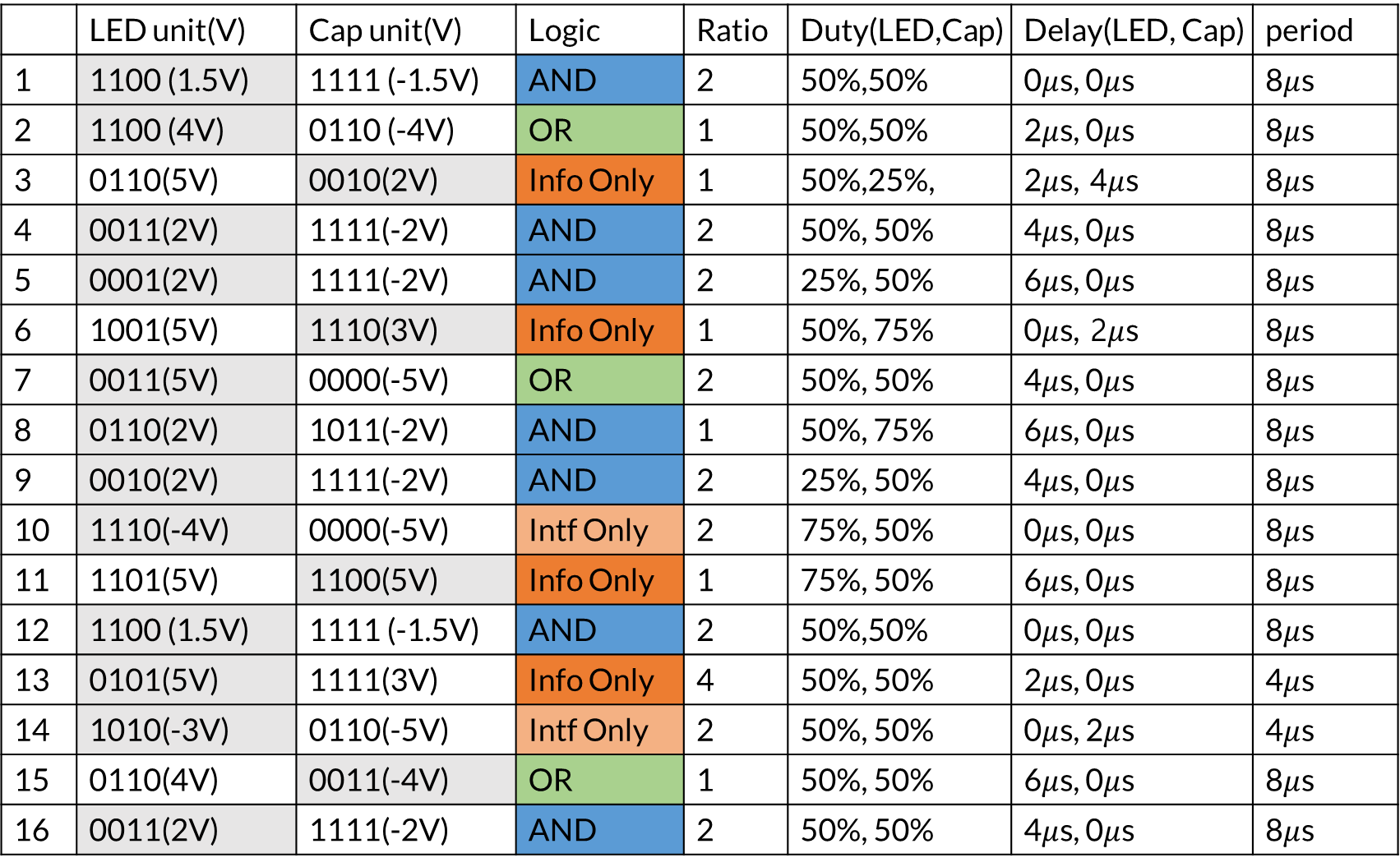
**

Figure S19. CEOI key for data decryption.

Supplementary Note 1.


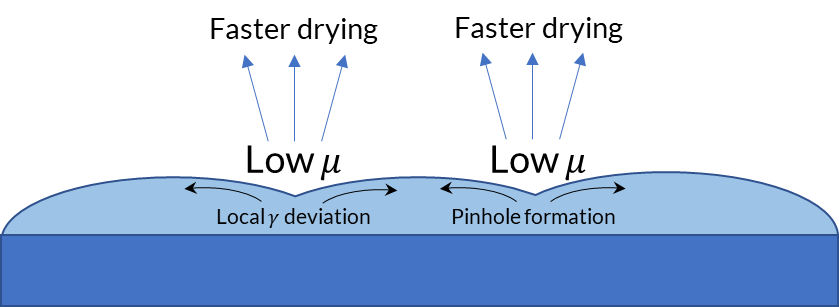


Figure S20. Schematic for pinhole formation by Bénard–Marangoni convection.

The co-solvent system of DMSO and ethanol promotes Bénard–Marangoni convection, which significantly contributes to pinhole formation by generating surface tension differences during vaporization. This effect can be characterized by the Marangoni number ($M_{a})$, which is defined as

$$M_{a}= \frac{-(\partial\gamma/\partial T)H^{2}\nabla T}{\mu\alpha}=\frac{-(\partial\gamma/\partial C)H^{2}\nabla C}{\mu D} ,$$

where $\gamma$ is the surface tension, *T* is the temperature, *C* is the concentration, *H* is the height of the solution layer, $\mu$is the viscosity, and $\alpha$ and $D$ are the thermal and mass diffusivities, respectively. Local surface tension differences induced by thermal or compositional gradients cause convective instability in the fluid. Consequently, the imbalance in surface tension caused by the differences in vapor pressure (VP) and viscosity between DMSO ($= \mu=2.14 mPa\cdot s, VP= 0.08 kPa)$ and ethanol ($\mu= 1.2mPa\cdot s, VP= 5.95kPa)$ leads to the formation of pinholes after the spin-coating process^[1, 2]^.

Supplementary Note 2.


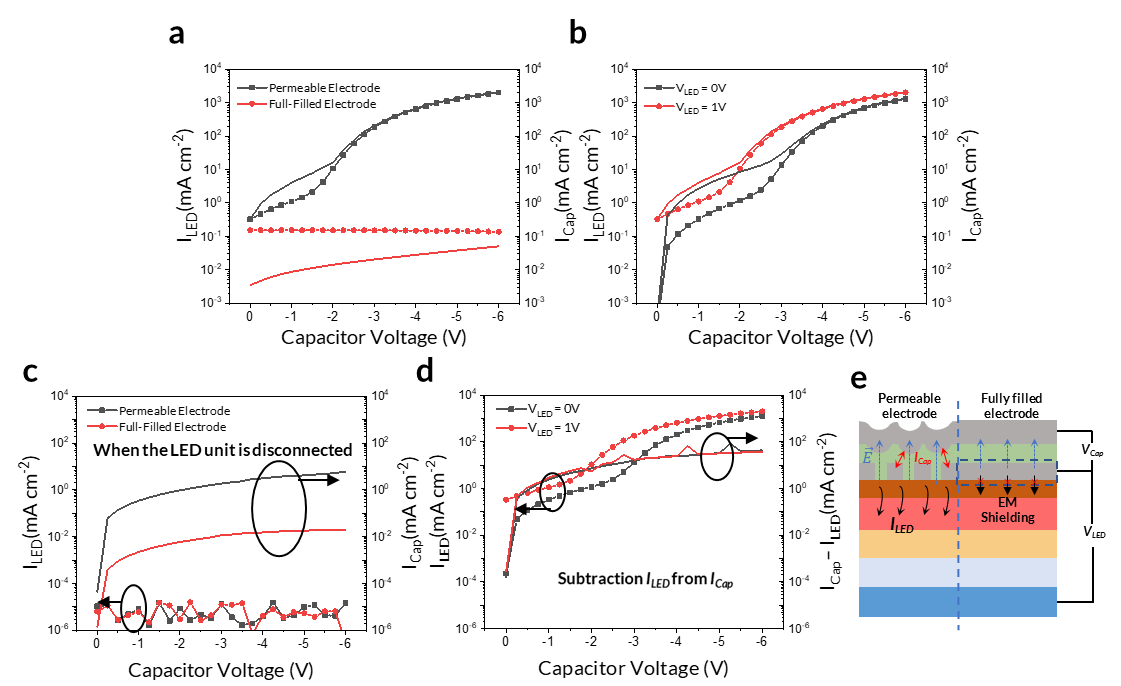


Figure S21 a, *I_LED_* and *I_Cap_* for permeable electrode and fully filled (i.e., solid, non-perforated) electrode at *V_LED_* = 0 V. b, Turn-on voltage shift by varying bias at LED unit (*V_LED_)* (lines with markers refer to I*_LED_*, and solid lines refer to I*_Cap._*). c, *I_LED_* and *I_Cap_* for permeable electrode and fully filled electrode when the LED unit is disconnected. d, Subtraction of *I_LED_* from *I_Cap_*. e. Schematic current flow illustration and operating image with permeable or fully filled electrode.

At the pinhole regions in the middle PE, the dielectric and Zn_87.5_Mg_12.5_O ETL make direct contact. Therefore, the capacitor unit in the PeLETs constructs parallel connections, with both metal–insulator–metal (Al/dielectric/Al, MIM) and metal–insulator–semiconductor (Al/dielectric/ETL, MIS) configurations. As a result, the current density of the capacitor unit, *I_Cap_*, represents the sum of the currents from both configurations (Supplementary Fig. 18a, b). This follows Kirchhoff’s current law, which states that the total current at a node is conserved, with the MIM and MIS currents adding up to *I_Cap_*.

However, the measured original *I_Cap_* does not indicate significant leakage current at the capacitor unit. Since the capacitor unit with PE consists of both MIM and MIS capacitors, the current passing through the PE and ETL interlayer appears to contribute to both *I_Cap_* and *I_LED_*. This can be supported by several experimental observations. First, as shown in Supplementary Fig. 18c, the *I_Cap_* measured with the LED unit disconnected (i.e., when the capacitor terminal at the top and the middle PE are only connected) closely corresponds to the current density obtained by subtracting the LED current (*I_LED_*) from the original *I_Cap_* (Supplementary Fig. 18d). This consistency was also observed across different *V_LED_*. In addition, the shift in *I_LED_* with varying *V_LED_* while the *I_Cap_ -* *I_LED_* values remain consistent, indicates that the LED unit modulation is driven by the E-field rather than leakage current from capacitor unit. Moreover, when operating with the Cap pulse, the TrEL response shows a delay compared to the response observed with the LED pulse (Fig. 3b-f), further supporting the E-field driven mechanism in our PeLETs.

The *I_Cap_* without the LED unit connected exhibits a difference between the PE and fully filled (non-perforated) electrode (Supplementary Fig. 18c). The increase in the *I_Cap_* is possibly attributed to the indented morphology caused by the PE as illustrated in Supplementary Fig. 18e. Both top electrode and dielectric may also be indented, resulting in the effective distance between PE and top electrode being shorter than in a fully filled electrode. However, this phenomenon could be overcome by optimizing the thickness of the dielectric and deposition method.

Supplementary Note 3.


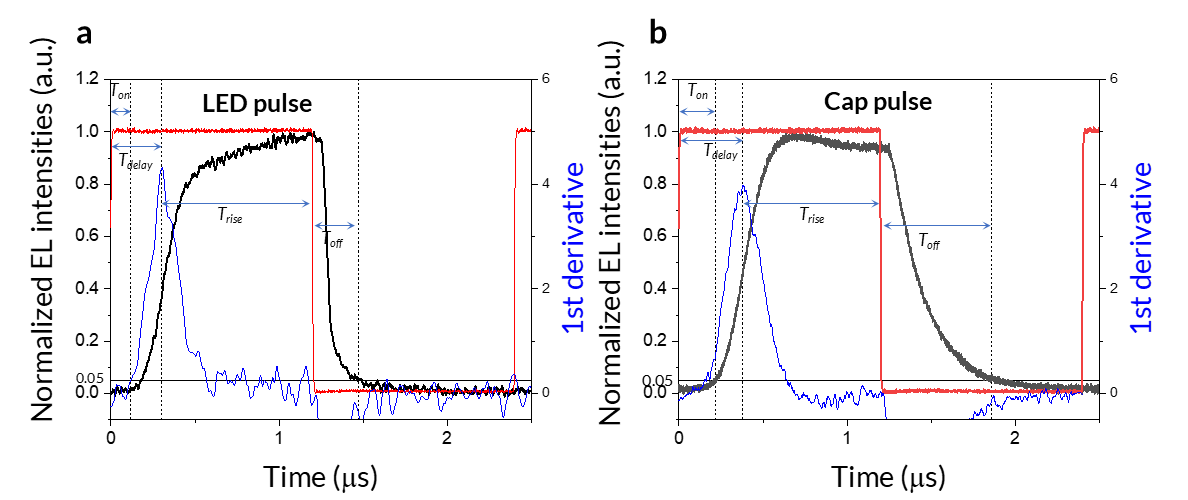


Figure S22 Transient EL waveforms under a, LED pulse (*V_LED_* = 5 V) and b, Cap pulse (*V_Cap_* = −5 V). Black curve: transient EL waveforms; red curve: voltage pulse; blue curve: first derivative of the transient EL waveforms.

The increase in EL induced by very short pulses follows several key factors during the brief blinking period. To clarify the physical meaning of carrier dynamics in PeLETs, we define several characteristic times (*T_delay_*, *T_rise_*, *T_off_*, and *T_on_*), as shown in Supplementary Fig. 19. In conventional LED, the driving forces for carrier recombination in the EML are drift and diffusion processes. At extremely short timescales following pulse stimulation, owing to the insufficient injection of carriers into the EML, the carriers in the device are affected predominantly by drift, as described by the Drude model (*J* = *neμV*/*d*, where *n* = carrier concentration, *e* = electric charge, *μ* = effective mobility of device, *V* = voltage, and *d* = thickness of EML). In the emission mechanism, the carrier flow is constrained by the EML thickness. The delay time, *t_d_*, follows the equation

$$t_{d}= \frac{d^{2}}{\left( \sqrt{\mu_{h}}+\sqrt{\mu_{e}} \right)^{2}V}\approx\frac{d^{2}}{\mu_{e}V} .$$

Thus, the physical meaning of the delay time (*T_delay_*) corresponds to the drift-dominant regime in the LED. After the drift-dominant regime, the carrier flow is influenced by an additional factor, diffusion, during the rising time (*T_rising_*). Furthermore, even in the off moment of the voltage, the remaining carriers persist in recombining via diffusion, and this diffusion-dominant emission regime is defined by *T_off_*. We also define an additional term, *T_on_*, to explain the intrinsic RC delay mechanism in the EL increment, which is biased by the capacitor unit. We extracted values that exceeded a threshold of 0.05. The rationale for selecting 0.05 was that the average noise level in our measurement setup was 0.025; therefore, we chose a threshold that was twice the average noise level. Therefore, the threshold for extracting the values of *T_on_* and *T_off_* was set to 0.05. Moreover, the boundary condition to separate *T_delay_* and *T_rising_* was determined by the time corresponding to the maximum of the first derivative of the EL increment^[3]^.

Supplementary Note 4.

The OOK bandwidth (Figure S13), which corresponds to -3 dB point under complete on-off switching, defines the minimum bit rate achievable in VLC systems, representing the ability to transmit one bit per time interval defined by the bandwidth.

In practical VLC applications, single-carrier signals are decomposed into multiple orthogonal subcarriers through discrete multitone (DMT) modulation, which enhances data transmission speed and spectral efficiency by enabling parallel data streams in the time domain. The orthogonality of these subcarriers is crucial for avoiding inter-channel interference and maintaining efficient modulation^[4]^. Therefore, the temporal response of carrier dynamics, characterized by the OOK bandwidth and TrEL measurements in PeLETs, plays a crucial role in achieving high-speed communication. Furthermore, colloidal QDs with narrow full-width-at-half-maximum (FWHM) emission are promising emissive materials for implementing multi-channel transmission in the spectral domain via wavelength division multiplexing (WDM) and via spatial separation using multiple-input multiple-output (MIMO)^[5, 6]^ techniques. These complementary VLC techniques collectively offer a promising pathway to significantly enhance the bit rate of PeLET-based systems.

In our work, we propose a device-level multi-channel transmission strategy based on the PeLET architecture. This approach opens a new dimension of control and system integration, offering a scalable platform for high-speed VLC and on-device data encryption, surpassing the limitations of conventional LED-based architecture

Supplementary Table S1. Overview of reported vertical three-terminal light-emitting devices and their key characteristics

| EML | PE | Peak (nm) | EQE (%) | CE  (cd/A) | PL lifetime | FWHM | Target application | ref |
| --- | --- | --- | --- | --- | --- | --- | --- | --- |
| CdSe | AgNW | Green  (~530 nm) | - | 37 | *~60 ns*^[7]^ | *~35 nm* | *Display* | ^[8]^ |
| Ir(MDQ)_2_  (acac) | Thin Al | Red  (~630 nm) | 19.6 | 20.6 | *>8* *μs*^[9]^ | *89.9 nm*^[9]^ | *Display* | ^[10]^ |
| Ir(ppy)_2_  (acac) | Thin Al | Green (~530 nm) | 24.6 | 90.1 | *>8* *μs*^[9]^ | *67.8 nm*^[9]^ | *Display* | ^[10]^ |
| Flrpic | Thin Al | Blue  (~ 470 nm) | 11.8 | 27.3 | *>8 μs*^[11]^ | *~65 nm* | *Display* | ^[10]^ |
| Ir(ppy)_2_  (acac) | Porous ITO | Green (~530 nm) | - | 13.1 | *>8 μs*^[9]^ | *67.8 nm*^[9]^ | *Display* | ^[12]^ |
| Ir(MDQ)_2_  (acac) | CNT | Red  (~630 nm) | - | ~12.4 | *>8 μs*^[9]^ | *89.9 nm*^[9]^ | *Display* | ^[13]^ |
| Ir(ppy)_2_  (acac) | CNT | Green (~530 nm) | - | ~49.8 | *>8 μs*^[9]^ | *67.8 nm*^[9]^ | *Display* | ^[13]^ |
| Flrpic | CNT | Blue  (~ 470 nm) | - | ~23.9 | *>8 μs*^[11]^ | *~65 nm* | *Display* | ^[13]^ |
| Ir(piq)_2_  (acac) | Thin Al | Red  (~622 nm) | - | 6 | *>8 μs*^[14]^ | *~80 nm* ^[14]^ | *Display* | ^[15]^ |
| Ir(mppy)_3_ | Thin Al | Green (~530 nm) | - | 26 | *>8 μs*^[16]^ | *~70 nm* | *Display* | ^[15]^ |
| Firpic | Thin Al | Blue  (~ 470 nm) | 11.8 | 27.3 | *>8 μs*^[11]^ | *~65 nm* | *Display* | ^[15]^ |
| InP | Perforated Al | Red  (633 nm) | 17.4 | 15.7 | ~40 ns^[7]^ | 38.6 nm | VLC | Our work |

*CE: Current Efficiency,

*The PL lifetimes of each emitter were extracted as average lifetimes from previously reported studies.

**Reference**

[1] Birnie, Dunbar P. III. 2013. *A Model for Drying Control Cosolvent Selection for Spin-Coating Uniformity: The Thin Film Limit*. Langmuir: 29. 9072–78. American Chemical Society. https://doi.org/10.1021/la401106z.

[2] Lee, Hanleem, William Harden-Chaters, Soo Deok Han, Shijie Zhan, Benxuan Li, Sang Yun Bang, Hyung Woo Choi, et al. 2020. *Nano-to-Microporous Networks via Inkjet Printing of ZnO Nanoparticles/Graphene Hybrid for Ultraviolet Photodetectors*. ACS Applied Nano Materials: 3. 4454–64. American Chemical Society. https://doi.org/10.1021/acsanm.0c00558.

[3] Keating, Logan P., Hyunho Lee, Steven P. Rogers, Conan Huang, and Moonsub Shim. 2022. *Charging and Charged Species in Quantum Dot Light-Emitting Diodes*. Nano Letters: 22. 9500–9506. American Chemical Society. https://doi.org/10.1021/acs.nanolett.2c03564.

[4] Khalid, A. M., G. Cossu, R. Corsini, P. Choudhury, and E. Ciaramella. 2012. *1-Gb/s Transmission Over a Phosphorescent White LED by Using Rate-Adaptive Discrete Multitone Modulation*. IEEE Photonics Journal: 4. 1465–73. https://doi.org/10.1109/JPHOT.2012.2210397.

[5] Lee, Wooram, Mahn Yong Park, Seung Hyun Cho, Jihyun Lee, C. Kim, Geon Jeong, and Byoung Whi Kim. 2005. *Bidirectional WDM-PON based on gain-saturated reflective semiconductor optical amplifiers*. IEEE Photonics Technology Letters: 17. 2460–62. https://doi.org/10.1109/LPT.2005.858148.

[6] Dang, Shuping, Osama Amin, Basem Shihada, and Mohamed-Slim Alouini. 2020. *What should 6G be?*. Nature Electronics: 3. 20–29. Nature Publishing Group. https://doi.org/10.1038/s41928-019-0355-6.

[7] Lee, Jaehwan, Jaeyeong Ha, Hyungdoh Lee, Hyunjin Cho, Doh C. Lee, Dmitri V. Talapin, and Himchan Cho. 2023. *Direct Optical Lithography of Colloidal InP-Based Quantum Dots with Ligand Pair Treatment*. ACS Energy Letters: 8. 4210–17. American Chemical Society. https://doi.org/10.1021/acsenergylett.3c01019.

[8] Chen, Qizhen, Yujie Yan, Xiaomin Wu, Shuqiong Lan, Daobing Hu, Yuan Fang, Dongxu Lv, Jianfeng Zhong, Huipeng Chen, and Tailiang Guo. 2019. *High-Performance Quantum-Dot Light-Emitting Transistors Based on Vertical Organic Thin-Film Transistors*. ACS Applied Materials & Interfaces: 11. 35888–95. American Chemical Society. https://doi.org/10.1021/acsami.9b11198.

[9] Liu, Yuan, Christian Hänisch, Zhongbin Wu, Paul-Anton Will, Felix Fries, Jinhan Wu, Simone Lenk, Karl Leo, and Sebastian Reineke. 2019. *Locking excitons in two-dimensional emitting layers for efficient monochrome and white organic light-emitting diodes*. Journal of Materials Chemistry C: 7. 8929–37. Royal Society of Chemistry. https://doi.org/10.1039/C9TC02768H.

[10] Wu, Zhongbin, Yuan Liu, Erjuan Guo, Ghader Darbandy, Shu-Jen Wang, René Hübner, Alexander Kloes, Hans Kleemann, and Karl Leo. 2021. *Efficient and low-voltage vertical organic permeable base light-emitting transistors*. Nature Materials: 20. 1007–14. Nature Publishing Group. https://doi.org/10.1038/s41563-021-00937-0.

[11] Hou, Liudong, Lian Duan, Juan Qiao, Deqiang Zhang, Liduo Wang, Yong Cao, and Yong Qiu. 2011. *Efficient solution-processed phosphor-sensitized single-emitting-layer white organic light-emitting devices: fabrication, characteristics, and transient analysis of energy transfer*. Journal of Materials Chemistry: 21. 5312–18. Royal Society of Chemistry. https://doi.org/10.1039/C0JM02987D.

[12] Yu, Hyeonggeun, Szuheng Ho, Nilesh Barange, Ryan Larrabee, and Franky So. 2018. *Semi-transparent vertical organic light-emitting transistors*. Organic Electronics: 55. 126–32. https://doi.org/10.1016/j.orgel.2018.01.030.

[13] McCarthy, M. A., B. Liu, E. P. Donoghue, I. Kravchenko, D. Y. Kim, F. So, and A. G. Rinzler. 2011. *Low-Voltage, Low-Power, Organic Light-Emitting Transistors for Active Matrix Displays*. Science: 332. 570–73. American Association for the Advancement of Science. https://doi.org/10.1126/science.1203052.

[14] Li, C.-L., Y.-J. Su, Y.-T. Tao, P.-T. Chou, C.-H. Chien, C.-C. Cheng, and R.-S. Liu. 2005. *Yellow and Red Electrophosphors Based on Linkage Isomers of Phenylisoquinolinyliridium Complexes: Distinct Differences in Photophysical and Electroluminescence Properties*. Advanced Functional Materials: 15. 387–95. https://doi.org/10.1002/adfm.200305100.

[15] Liu, Jiang, Dustin Chen, Xinning Luan, Kwing Tong, Fangchao Zhao, Chao Liu, Qibing Pei, and Huaping Li. 2017. *Electrolyte-Gated Red, Green, and Blue Organic Light-Emitting Diodes*. ACS Applied Materials & Interfaces: 9. 12647–53. American Chemical Society. https://doi.org/10.1021/acsami.7b00463.

[16] Fukagawa, Hirohiko, Takahisa Shimizu, Taisuke Kamada, Shota Yui, Munehiro Hasegawa, Katsuyuki Morii, and Toshihiro Yamamoto. 2015. *Highly efficient and stable organic light-emitting diodes with a greatly reduced amount of phosphorescent emitter*. Scientific Reports: 5. 9855. Nature Publishing Group. https://doi.org/10.1038/srep09855.
